# CO_2_ electrochemical catalytic reduction with a highly active cobalt phthalocyanine

**DOI:** 10.1038/s41467-019-11542-w

**Published:** 2019-08-09

**Authors:** Min Wang, Kristian Torbensen, Danielle Salvatore, Shaoxuan Ren, Dorian Joulié, Fabienne Dumoulin, Daniela Mendoza, Benedikt Lassalle-Kaiser, Umit Işci, Curtis P. Berlinguette, Marc Robert

**Affiliations:** 1 Université de Paris, Laboratoire d’Electrochimie Moléculaire, CNRS, F-75013 Paris, France; 2 Department of Chemical and Biological Engineering, The University of British Columbia, 2360 East Mall, Vancouver, BC V6Y 1Z3 Canada; 3 Department of Chemistry, The University of British Columbia, 2036 Main Mall, Vancouver, BC V6T 1Z1 Canada; 4 Gebze Technical University, Department of Chemistry, 41400 Gebze, Kocaeli Turkey; 5 Stewart Blusson Quantum Matter Institute, The University of British Columbia, 2355 East Mall, Vancouver, BC V6T 1Z4 Canada; 6 Synchrotron SOLEIL, L’Orme des Merisiers, Saint-Aubin, 91192 Gif-sur-Yvette, France

**Keywords:** Electrocatalysis, Catalyst synthesis, Carbon nanotubes and fullerenes, Electrocatalysis

## Abstract

Molecular catalysts that combine high product selectivity and high current density for CO_2_ electrochemical reduction to CO or other chemical feedstocks are urgently needed. While earth-abundant metal-based molecular electrocatalysts with high selectivity for CO_2_ to CO conversion are known, they are characterized by current densities that are significantly lower than those obtained with solid-state metal materials. Here, we report that a cobalt phthalocyanine bearing a trimethyl ammonium group appended to the phthalocyanine macrocycle is capable of reducing CO_2_ to CO in water with high activity over a broad pH range from 4 to 14. In a flow cell configuration operating in basic conditions, CO production occurs with excellent selectivity (ca. 95%), and good stability with a maximum partial current density of 165 mA cm^−2^ (at −0.92 V vs. RHE), matching the most active noble metal-based nanocatalysts. These results represent state-of-the-art performance for electrolytic carbon dioxide reduction by a molecular catalyst.

## Abstract (web summary)

Molecular electrocatalysts reducing CO_2_ to CO with high selectivity and high rate are urgently needed. A cobalt phthalocyanine complex is capable of reducing CO_2_ to CO in water with a maximum partial current density up to 165 mA cm^−2^, matching the most active noble metal-based nanocatalysts.

## Introduction

CO_2_ can potentially be used as a renewable feedstock in electrochemical devices for sustainable energy storage in the form of synthetic fuels or fuel precursors (such as CO, CH_3_OH, and CH_4_), or for commodity chemicals. Hence, electrochemical reduction of CO_2_ is an attractive way for industry to exploit surplus sustainable electricity in periods of low demand and it may contribute to mitigating the environmental impact caused by the massive release of CO_2_^1–3^. However, the efficient and robust electrolytically driven reduction of CO_2_ remains a formidable challenge for the scientific community. Fundamentally new approaches are needed to realize the selective transformation of CO_2_ into desired products in an industrial setting.

There are several approaches available to catalyze the carbon dioxide reduction reaction (CO_2_RR), including molecular catalysts^4–7^. Molecular transition metal catalysts offer the distinct advantage of allowing for the fine-tuning of the primary and secondary coordination spheres by manipulating the chelating environment and the steric and electronic effects of the ligands. The ability to improve catalytic efficiency and product selectivity through the rational optimization of the ligand structure is a feature not accessible to the solid-state catalysts common to pilot-scale electrolyzer units^8,9^.

There now exist a range of known molecular catalysts, including those based on noble (e.g., Ru, Ir, and Re) and earth-abundant metals (e.g., Co, Ni, Fe, Mn, and Cu)^4–12^. These catalysts typically mediate a two electron reduction of CO_2_ to either CO or formate with reasonable efficiencies, but in organic solvents. (there are also rare reports demonstrating molecules to catalyze highly reduced products such as light hydrocarbons)^13–15^. Integrating molecules with thin porous carbon films, such as carbon powder, carbon nanotubes, or graphene to form hybrid catalytic materials has proven to be a promising strategy to selectively achieve CO production in pure aqueous conditions. Importantly, these systems used earth-abundant metal catalysts such as Fe and Co porphyrins^16–23^, Mn bipyridine complexes^24,25^, Co quaterpyridines^26^, and Co phthalocyanines^27–32^. Good performances have been obtained in close to neutral conditions (pH 7–7.5) with excellent selectivity. The state-of-the art system is a Co quaterpyridine adsorbed onto multiwalled carbon nanotubes, which shows exceptional selectivity (99%) at a current density up to 20 mA cm^−2^ at a 440 mV overpotential^26^. An octacyano-substituted Co phthalocyanine complex deposited onto a gas diffusion electrode (GDE) and tested in a flow cell has been reported to achieve a current density of ~30 mA cm^−2^ at an overpotential of ~550 mV with 96% selectivity for 10 h^31^.

While these values represent important advances for molecular CO_2_RR catalysts, much higher current densities are needed for commercial operation. Moroever, these current densities remain far below those obtained with state-of-the-art solid-state Ag^33,34^ or Au^35^ nanomaterials that have been reported to reach >150 mA cm^−2^.

In this work, we design a new Co phthalocyanine (**CoPc2**) bearing one trimethyl ammonium moiety and three *tert*-butyl groups appended on the phthalocyanine macrocycle. The cobalt complex is obtained and used as a mixture of the different regioisomers, which exhibit identical electronic and steric properties^36,37^, and **CoPc2** thus gives a unique electrochemical signature (Supplementary Fig. 1) with three reversible Faradaic waves that could be assigned to the Co^II^/Co^I^ redox couple and to ligand reduction peaks^38,39^. With relatively low catalyst loadings of **CoPc2** dispersed into porous films of carbon black powder or carbon nanotubes on a carbon paper cathode, the modified electrode functions as highly effective CO_2_RR electrocatalysts in acidic (pH 4), neutral (pH 7.3), and basic conditions (pH 14). Importantly, the conversion of CO_2_ to CO with very high selectivities and current densities (up to 165 mA cm^−2^ at pH 14) closely matches the performances of the most active noble metal-based catalysts.

## Results

### Preparation and electrochemical characterization of the catalytic inks

**Fig. 1:**
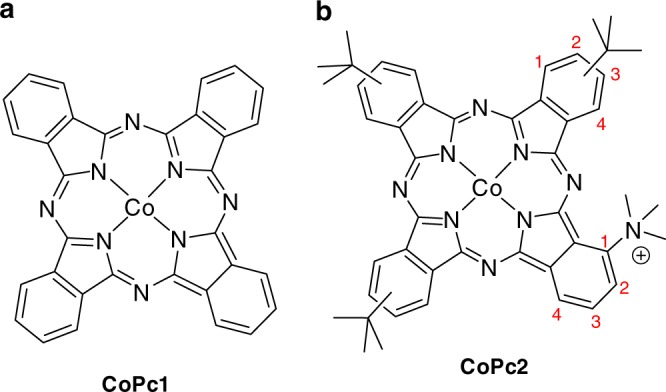
Cobalt phthalocyanine catalysts **CoPc1** and **CoPc2** investigated in this study. **a** **CoPc1** bears no substituents. **b** **CoPc2** bears one trimethyl ammonium group at position 1 of the isoindole subunits, and three *tert*-butyl groups (positions 2 or 3) of the other subunits

Cobalt complexes **CoPc1** (Fig. 1a) and **CoPc2** (Fig. 1b) were deposited onto carbon electrodes from prepared colloidal inks.

**Fig. 2:**
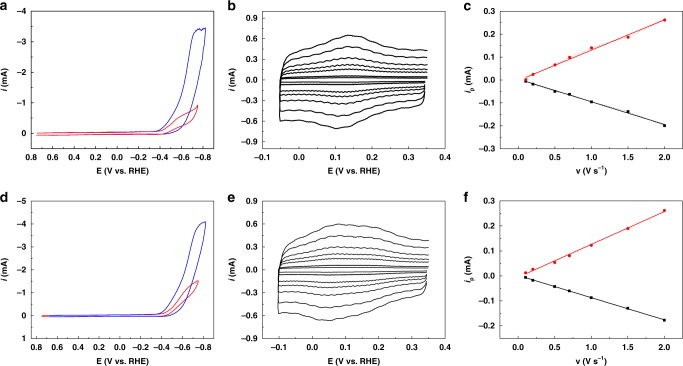
Cyclic voltammetry of cobalt phthalocyanine catalytic films. Top **a**–**c** **CoPc1**@MWCNTs, bottom **d**–**f** **CoPc2**@MWCNTs. **a**, **d** CV of a cobalt phthalocyanine catalytic film deposited onto a glassy carbon electrode (*d* = 3 mm) in 0.5 M NaHCO_3_ at *v* *=* 0.1 V s^−1^ under argon (red, pH 8.5) and CO_2_ (blue, pH 7.3). **b**, **e** CV at various scan rates of the Co^II^/Co^I^ wave under argon atmosphere. **c**, **f** Variation of the peak current vs. scan rate for the Co^II^/Co^I^ redox wave. A redox active catalyst concentration of *Γ* = 1.45 ± 0.12 nmol cm^−2^ was obtained from *i*_p_ = *n*^2^*F*^2^*vSΓ*/4*RT* for both catalysts

Briefly, multiwalled carbon nanotubes (MWCNTs) or carbon black were dispersed in 1:1 mixtures of EG and ethanol by sonication. The catalyst was then added to the suspension, and after sonication and addition of a small amount of Nafion^®^, the catalytic material was drop casted on a glassy carbon electrode (*d* = 3 mm) for cyclic voltammetry (CV) characterization, or onto carbon paper (1 or 0.5 cm^2^) for preparative scale electrolysis. Typical CVs recorded in a 0.5 M NaHCO_3_ aqueous solution (pH 7.3) are shown in Fig. 2. The reversible wave centered at ca. −0.1 V vs. RHE could be assigned to the surface confined Co^II^/Co^I^ redox couple, as shown by the linear increase of the peak current as a function of the scan rate (Fig. 2b, e).

 The electroactive amount of catalyst within the film was 1.3–1.5 nmol cm^−2^, corresponding to about 9% of the total loaded concentration (Fig. 2e, f). Upon CO_2_ saturation of the solution, the Co^II^/Co^I^ wave remained unchanged, while a large catalytic increase of the current occurs at potentials more negative than −0.4 V vs. RHE (Fig. 2a, d). Repetitive CV shows good stability of the current response, indicative of a stable catalytic film (Supplementary Fig. 2). Remarkably, **CoPc2** gives a larger catalytic current at potentials very close from **CoPc1**, indicative of a better activity on kinetic grounds. We conjecture that the enhanced reactivity of **CoPc2** for CO_2_ to CO conversion may be attributed to the through-space interactions between the positive charge of the trimethyl ammonium substituent and the partial negative charge borne by the O atoms in CO_2_, which facilitate the reductive coordination of the CO_2_ molecule to the Co metal center. This mechanism has been reported for a tetraphenyl Fe porphyrins substituted with same functional groups on the phenyl rings for CO_2_ to CO conversion^8^. These interactions may also favor the subsequent C–O bond cleavage leading to CO formation, thus illustrating how simple tuning of the phthalocyanine substituents can accelerate the catalytic reaction. Further mechanistic studies are in preparation.

### Electrolysis experiments at neutral and acidic pH

**Fig. 3:**
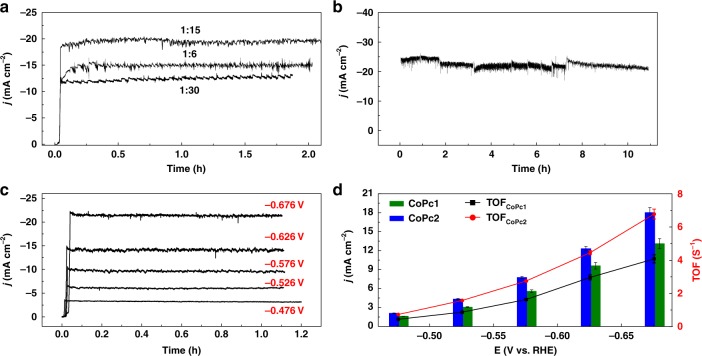
Controlled potential electrolysis of CO_2_ reduction. **a** Electrolysis current densities (*E* = −0.676 V vs. RHE) for a **CoPc2**@MWCNTs film at various catalyst mass ratio. **b** Long-term electrolysis (*E* = −0.676 V vs. RHE) at optimized mass ratio. **c** Variation of the current as a function of the electrolysis potential at optimized mass ratio (see text). **d** Current density and rate constant (TOF) for **CoPc1**@MWCNTs and **CoPc2**@MWCNTs for CO production in a CO_2_ saturated solution containing 0.5 M NaHCO_3_ (pH 7.3). The uncertainties represent standard errors obtained from four measurements

After the catalytic ink was deposited on a carbon paper electrode (see Methods and Supplementary Fig. 3 for a scanning electron microscopy (SEM) image of the porous catalytic film), bulk electrolysis was performed at pH neutral conditions with **CoPc2** for various loadings of the molecular catalyst. An optimal loading ratio of 1:15 (catalyst:MWCNTs weight ratio) shows reasonably good stability over the course of the 2 h experiments (Fig. 3a).

The CO selectivity was typically 92–93% in repeated runs, with H_2_ being identified as a minor gas phase by-product (7–8%). No other product was detected in the liquid phase upon nuclear magnetic resonance (NMR) analysis. The electrolysis performed in the absence of catalyst or with **CoPc2**@MWCNTs (1:15) under Ar atmosphere only furnished H_2_ (Supplementary Fig. 4). The TON and TOF values in Tables 1 and 2 and in the text were calculated using the total amount of catalyst added in the films, hence these values are underestimated.

**Table 1:** Comparative data for **CoPc1**@MWCNTs and **CoPc2**@MWCNTs catalytic materials during a 1 h electrolysis as a function of the potential in CO_2_-saturated aqueous solution containing 0.5 M NaHCO_3_ (pH 7.3)

*E* (V vs. RHE)	Entry	overpotential (mV)^a^	Catalyst^b^	*j*_CO_ (mA cm^−2^)^c^	TOF (CO, s^−1^)^c^	Selectivity (CO, ±0.5%)
−0.676	1	546	**CoPc1**	13.10 ± 0.79	4.08 ± 0.25	92
	2	539	**CoPc2**	18.10 ± 0.80	6.81 ± 0.30	93
−0.626	3	502	**CoPc1**	9.61 ± 0.51	2.97 ± 0.16	92
	4	498	**CoPc2**	12.3 ± 0.34	4.46 ± 0.12	92
−0.576	5	458	**CoPc1**	5.57 ± 0.21	1.63 ± 0.06	86
	6	454	**CoPc2**	7.75 ± 0.16	2.75 ± 0.06	84
−0.526	7	411	**CoPc1**	3.10 ± 0.07	0.87 ± 0.02	80
	8	410	**CoPc2**	4.33 ± 0.08	1.57 ± 0.03	73
−0.476	9	364	**CoPc1**	1.62 ± 0.01	0.44 ± 0.01	76
	10	363	**CoPc2**	2.09 ± 0.04	0.74 ± 0.01	65

^a^ Corrected from ohmic drop (uncompensated solution resistance of ca. 3 Ω, electrode surface 0.5 cm^2^)

^b^*Γ*(**CoPc1**) = 23.3 nmol cm^−2^, *Γ*(**CoPc2**) = 14.4 nmol cm^−2^ (total added catalysts)

^c^The uncertainties represent standard errors obtained from four measurements

**Table 2:** Comparison of electrolysis performances between **CoPc2**@carbon powder hybrid catalyst and previously reported state-of-the art immobilized molecular Co catalysts and Ag nanomaterial

Entry	Catalyst	*E* (V vs. RHE) [overpotential(mV)]	Electrolyte	*j*_CO_ (mA cm^−2^)	TOF (s^−1^)	CO sel. (%)	Cell type	Ref.
**1**	**CoPc2**	−0.97 [836]^a^	0.5 M KCl	16.3	6.1	92	H cell	this work
**2**	**CoPc2**	−0.676 [539]^a^	0.5 M NaHCO_3_	18.1	6.8	93	H cell	this work
**3**	**CoPc**	−0.676 [546]^a^	0.5 M NaHCO_3_	13.1	4.1	92	H cell	this work
**4**	CoPc-CN	−0.63 [520]	0.1 M KHCO_3_	14.7	4.1	98	H cell	28
**5**	CoPpc	−0.61 [500]	0.5 M NaHCO_3_	18	1.4	ca. 90	H cell	29
**6**	Coqpy	−0.55 [440]	0.5 M NaHCO_3_	19.9	12	99	H cell	26
**7**	**CoPc2**	−0.31 [200]^b^	1 M KOH	22.2	0.54	93	Flow cell	this work
**8**	**CoPc2**	−0.65 [540]^b^	1 M KOH	70.5	1.67	94	Flow cell	this work
**9**	**CoPc2**	−0.72 [610]^b^	1 M KOH	111.6	2.7	96	Flow cell	this work
**10**	**CoPc2**	−0.92 [810]^b^	1 M KOH	165	3.9	94	Flow cell	this work
**11**	CoPc-CN	−0.66 [550]	1 M KOH	31	/	94	Flow cell	31
**12**	Ag^c^	−0.81 [700]	1 M KOH	156.5	/	92	Flow cell	34

Entry 1: *Γ* = 14.4 nmol cm^−2^ (pH 4, 2 h electrolysis), entry 2: *Γ* = 14.4 nmol cm^−2^ (pH 7.3, 1 h electrolysis), entry 3: *Γ* = 23.3 nmol cm^−2^ (pH 7.3, 1 h electrolysis), entries 7–10: *Γ* = 0.216 μmol cm^−2^ (pH 14, for 0.5, 10, 3, and 0.3 h electrolysis, respectively).

^a^Corrected from ohmic drop (uncompensated solution resistance of ca. 3 Ω, electrode surface 0.5 cm^2^)

^b^Uncorrected from ohmic drop

^c^Carbonate-derived Ag nano-catalyst (500 nm thickness), see Ref. ^34^ for details

Using this 1:15 catalyst loading weight ratio, the effect of the applied potential on efficiency and selectivity was investigated for both the cobalt catalysts at pH neutral conditions (Fig. 3c and Supplementary Fig. 5). As shown in Fig. 3d, the current density was systematically larger for **CoPc2** as compared to **CoPc1** (ca. 25% increase). Concomitantly, the CO selectivity increases at more negative electrolysis potentials, with a relative higher increase for **CoPc2** (Table 1). This shows that, at more negative potentials, the catalytic CO_2_ reduction reaction outruns the H_2_ evolution reaction. At −0.676 V vs. RHE which corresponds to an overpotential *η* = 546 mV and in neutral pH conditions, **CoPc1** gave 92% CO with an average partial current density of 13.1 mA cm^−2^ (Table 1, entry 1). At the same potential (*η* = 539 mV), a 1 h electrolysis with **CoPc2** led to a TON of 24516 for CO (TOF = 6.8 s^−1^) with 93% selectivity. The partial current density for CO production reaches 18.1 mA cm^−2^ (Table 1, entry 2).

Longer term electrolysis at pH 7.3 further demonstrates the excellent stability of the catalytic system, since no decrease in either the current density or the selectivity for CO production were observed upon a 10.5 h experiment. An average selectivity of 91% for CO production along with *j*_CO_ close to 20 mA cm^−2^ (Fig. 3b) were measured. XPS analysis of the cathode material before and after electrolysis does not reveal major changes of the Co 2p and N 1 s peaks, suggesting integrity of the molecular catalyst during the entire course of the electrolysis (Supplementary Fig. 6). Remarkably, an electrolysis (2 h) at *E* = − 0.971 V vs. RHE in a CO_2_ saturated 0.5 M KCl solution (pH 4) led to equally good results, with a selectivity of 92% and *j*_CO_ = 17 mA cm^−2^ (Supplementary Fig. 7). When the cobalt phtalocyanine bearing four t-butyl groups (**4-Co**) was used as catalyst in an electrolysis experiment (1:15 catalyst loading weight ratio, *E* = −0.676 V vs. RHE) in neutral pH conditions, a Faradaic efficiency of 92% for CO was obtained with an average CO partial current density of 15.8 mA cm^−2^ (Supplementary Fig. 8). This reference complex is thus less active than **CoPc2**, further illustrating that the enhanced activity of **CoPc2** may arise from the trimethyl ammonium substituent on the ligand via through-space effects.

### Electrolysis experiments in basic solutions using a flow cell

**Fig. 4:**
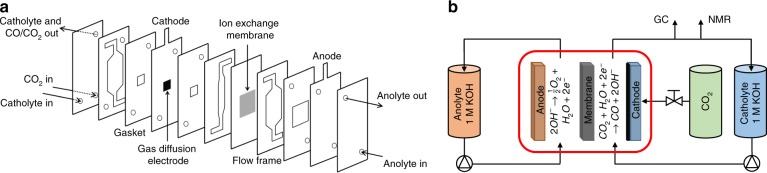
Graphic illustration of the CO_2_ electrolyzer flow cell. **a** Cross-sectional view of the cell and **b** general scheme of the entire experimental set-up

Encouraged by these results, we included **CoPc2** into a flow cell setup comprising **CoPc2** supported on a GDE as the cathode, in order to realize CO_2_ to CO conversion in industrial setting. Details of the setup are provided in the methods. Briefly, the electrolyzer consists of a sandwich of flow frames, electrodes, gaskets and an ion exchange membrane, which were assembled as schematically illustrated in Fig. 4a. A gas flow of CO_2_ is delivered from the back side of the cathodic compartment and flows through the GDE, while the catholyte solution is circulated in between the GDE and the anion exchange membrane (AEM). On the other side of the AEM, the anolyte is directed between the AEM and the Pt/Ti alloy anode, Fig. 4b. **CoPc2** was dispersed in a colloidal ink with carbon black and subsequently deposited on the carbon fiber paper, composing the cathode.

**Fig. 5:**
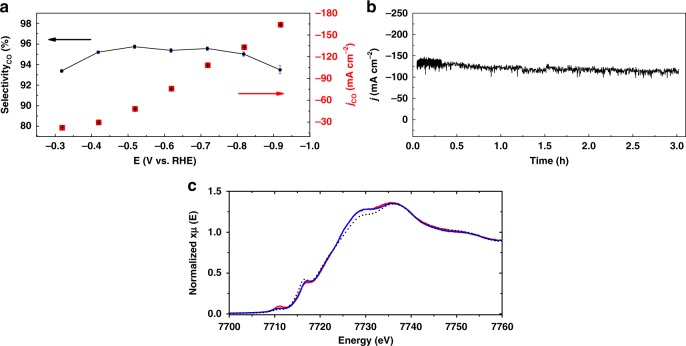
Controlled potential electrolysis and **CoPc2** film characterization. **a** Current density and selectivity for CO production as a function of the potential and **b** bulk electrolysis at fixed potential (*E* *=* −0.72 V vs. RHE) for **CoPc2**@carbon black deposited onto a carbon paper as cathodic material, in 1 M KOH. **c** Co K-edge XANES profiles of CoPc2 (black dots) and **CoPc2**@carbon black before (blue) and after electrolysis (*E* *=* − 0.72 V vs. RHE) (red) in 1 M KOH solution

At −0.3 V vs. RHE, which corresponds to a low 200 mV overpotential, a high current density with *j*_CO_ = 22.2 mA cm^−2^ was achieved. The dependence of the current density for CO production is reported in Fig. 5a (see also Supplementary Fig. 9). Upon setting the electrolysis potential at −0.72 V vs. RHE, *j*_CO_ raised to 111.6 mA cm^−2^ with 96% selectivity, while excellent stability over the course of the 3 h electrolysis was obtained (Fig. 5b). The only additional gas phase by-product was H_2_ (4% selectivity) and the catholyte solution was carefully checked by ^1^H NMR. Formate or methanol were not detected while the absence of any **CoPc2** trace further indicate that the molecular catalyst is not leaching out of the supporting film. The obtained *j*_CO_ corresponds to a turnover frequency (TOF) of 2.7 s^−1^ and a turnover number (TON) of 29,008. A maximum current density of 165 mA cm^−2^ for CO generation was obtained at −0.92 V vs. RHE. Long-term stability of the catalytic material was further illustrated upon applying a constant current density of 75 mA cm^−2^ for 10 h, which led to a cathodic potential of −0.65 V vs. RHE (*η* = 540 mV) with 94% selectivity of CO (*j*_CO_ = 70.5 mA cm^−2^) (Supplementary Fig. 10). All the collected data are compiled in Table 2, along with a comparison of previously reported catalysts. The type of cell used (H cell vs. flow cell) is also indicated to ease comparison between data. The Co K-edge XANES spectra of **CoPc2**@carbon black were recorded before and after electrocatalysis at *E* = −0.72 V vs. RHE. Figure 5c shows these spectra together with that of the starting **CoPc2** complex. All these spectra present the typical features expected for a cobalt (II) phthalocyanine complex, i.e., a low intensity pre-edge peak at 7711 eV (corresponding to a 1s to 3d/4p transition) and a shoulder at 7717 eV (corresponding to a 1s to 4p_z_ transition)^29,40,41^. The intensity of these two transitions were shown by Li et al.^41^ to depend on the attachment to a surface, i.e., the pre-edge intensity increases and the shoulder decreases upon adsorption onto nanotubes, respectively.

The same trend is observed in the **CoPc2**@carbon black system, with decreased pre-edge and increased shoulder intensities on going from the starting complex to the adsorbed species. This trend retains after catalysis, suggesting an even closer interaction with the surface while maintaining the overall structure of the molecular catalyst after the experiment. This conservation of structure is further confirmed by the EXAFS spectra (Supplementary Fig. 11), which also present the typical features of a cobalt phthalocyanine complex^41^. In addition, comparison of the spectrum of **CoPc2**@carbon black recorded after catalysis with those of reference cobalt samples (Supplementary Fig. 11) clearly shows that the changes observed on the spectrum after catalysis are insignificant as far as the overall structure is concerned.

## Discussion

Remarkably, **CoPc2** remains highly selective for the CO_2_-to-CO conversion across 10 pH units, extending from acidic (pH 4) to basic solutions (pH 14). An averaged 92% selectivity for CO_2_ reduction with partial current densities of ca. 20 mA cm^−2^ were routinely obtained in the whole domain of pH values with excellent stability over time. In close to neutral solutions (pH 7.3), **CoPc2** is a significantly better catalyst than the nonsubstituted phthalocyanine **CoPc1** (see Table 2, entries 2 and 3) with a ca. 25% increase in current density at similar overpotential, but it also surpasses state-of-the art tetra-cyano substituted phthalocyanine (CoPc-CN, Table 2, entry 4) and unsubstituted Co phthalocyanine polymerized around carbon nanotubes (CoPpc, Table 2, entry 5), both in terms of current density and TOF. Similarly to the previously reported cobalt phthalocyanines mentioned above, **CoPc2** exhibits excellent stability over time, as illustrated in Fig. 3b, showing a 10.5 h electrolysis experiment, with no loss of performance. A TOF up to 6.8 s^−1^ was reached and, generally, only a very small loading of the catalysts was necessary to obtain high *j*_CO_ (see for example Table 2, entries 1–2). In a 1 M KOH electrolyte solution (pH 14), ca. 20 mA cm^−2^ could be obtained at a very low overpotential of 200 mV, once the hybrid catalyst mixed with carbon support was included in a gas flow cell (Table 2, entry 7). Long-term electrolysis (10 h) in basic conditions (pH 14) at −0.65 V vs. RHE led to an average *j*_CO_ close to 70.5 mA cm^−2^. The ability to implement **CoPc2** in various pH conditions is also a key feature that may allow for combining the Co catalyst to various types of anodic materials in order to decrease the overall cell potential. In particular, the excellent performance obtained at pH 14 could permit pairing of the **CoPc2** loaded cathode with the most efficient oxygen evolving metal oxide anode materials. At this pH, at −0.92 V vs. RHE, this hybrid catalyst can form CO with 94% selectivity at a partial current density of 165 mA cm^−2^, matching the state-of-the art Ag based catalyst sputtered onto a PTFE membrane, both in terms of selectivity and current density (Table 2, compare entries 10 and 12).

In conclusion, upon introducing a positively charged trimethyl ammonium group and three tert-butyl groups on the parent cobalt phthalocyanine, a highly efficient and versatile catalyst for the CO_2_-to-CO electrochemical conversion in water has been obtained. This study highlights that rational tuning of the structure of simple metal complexes may allow for high CO_2_ electroreduction performances, and it is likely that further improvement is yet to come. This work opens up new perspectives for the development of low-cost catalytic materials to be included in CO_2_ electrolyzers that will hopefully soon emerge at an industrial scale.

## Methods

### Chemicals

Chemicals and materials were purchased from Sigma-Aldrich, Fluka, TCI America, ABCR or Alfa Aesar, and used as received. All aqueous solutions were prepared with Millipore water (18.2 MΩ cm). The MWCNTs were purchased from Sigma-Aldrich (O.D. × L 6–9 nm × 5 μm, >95%). The cobalt (II) phthalocyanine (**CoPc1**) (β-form, dye content 97%) was purchased from Sigma-Aldrich. Toray Carbon Paper (CAS number: 7782-42-5), TGP-H-60, 19 × 19 cm was purchased from Alfa Aesar and used for preparation of the cathodes for the electrochemical cell. The cathodes (GDEs) used in the flow cell were prepared using Freudenberg C24H5 carbon paper (21 × 29.7 cm, product code F5GDL). VULCAN^®^ XC72R Speciality Carbon Black was purchased from Cabot Corporation. 4-*Tert*-butylphthalonitrile was obtained from TCI America. 3-Nitrophthalonitrile was purchased from ABCR. All solvents were of synthetic grade. Supplementary Fig. 13 describes the synthesis and characterization of **CoPc2**, and supplementary Figs. 14–27 provide characterization of reaction intermediates and final compound.

### Preparation of the hybrid materials

For CV experiments and electrolysis in the closed electrolysis cell, 3 mg of MWCNTs were dispersed in 2 mL ethylene glycol (EG)/ethanol (EtOH) 1:1(v/v) mixture followed by 30 min of sonication. Totally, 1 mg of the cobalt catalytst (**CoPc1**, **CoPc2**) was dissolved in 1 mL EG/EtOH mixture. Various volumes of this solution were added to the MWCNTs suspension in a total volume of 3 mL, so as to get mass ratio (1:6, 1:15 and 1:30) of the catalyst. The suspension was further sonicated for 30 min. Finally, Nafion^®^ was added (2.9 %, 30 μL) and the complete mixture was sonicated for 30 min to obtain the final catalytic ink.

For the flow cell set-up, 3 mg of carbon black were dispersed in 3 mL EtOH followed by 30 min of sonication. Totally, 0.2 mg of **CoPc2** was dissolved in 1 mL EtOH so as to get a mass ratio (1:15) of the catalyst. The suspension was further sonicated for 30 min. Finally, Nafion^®^ was added (2.9%, 30 μL) and the complete mixture was sonicated for 30 min to obtain the final catalytic ink. The ink was drop casted on carbon paper masked with a Teflon frame to obtain an electrode area of 1 × 1 cm^2^.

### Electrochemical studies

CV experiments were performed using an AUTOLAB PGSTAT128N potentiostat (Metrohm). Controlled potential electrolysis were performed using a PARSTAT 4000A potentiostat (Princeton Applied Research).

*CV*: The three-electrode setup consisted of a glassy carbon working electrode (custom made, 0.071 cm^2^), a Pt wire counter electrode, and a SCE reference electrode (−0.241 V vs. NHE). The working electrode was polished with diamond paste (15, 6, 3, and 1 μm successively, 60 s per polishing cycle), thoroughly rinsed and sonicated in ethanol, and dried. Totally, 10 μL of the hybrid materials suspension were dropped on the surface of the electrode, and allowed to dry under 100 °C ambient conditions. Ohmic drop was compensated using the positive feedback compensation implemented in the instrument.

*Preparative scale electrolysis*: In the closed cell, experiments were carried out in a cell using a Toray carbon paper as working electrode, and a SCE reference electrode closely positioned one from the other. The Pt grid counter electrode was separated from the cathodic compartment with a glass frit. The catalytic ink was dropped on one face of the Toray carbon paper cathode (100 μL for a 0.5 cm^2^ electrode), and allowed to dry under 100 °C ambient conditions prior to use. The full cell setup was identical to the one used previously^26^.

The flow cell electrolyzer (Micro Flow Cell^®^ purchased by Electrocell) is composed by a sandwich of flow frames, electrodes, gaskets, and a membrane, which, when assembled as illustrated in Fig. 3, constitute a three-compartment flow cell. One compartment delivers the CO_2_ (at 16.7 sccm) from the back side and through the GDE (1 × 1 cm^2^, fixated in a Ti frame), while another directs the catholyte solution (1 M KOH, flow rate of 16 sccm) in between the GDE and the AEM (Sustainion^TM^ X37-50). On the other side of the latter, the anolyte (1 M KOH, flow rate of 16 sccm) is directed between the AEM and the Pt/Ti alloy anode. The flow frames are made of PTFE, and the gaskets of peroxide cured EDPM. Catholyte and anolyte were recycled using peristaltic pumps. All tubings were made of PTFE and connected to the cell with PEEK ferrules and fittings. The whole setup is schematically shown Supplementary Fig. 12.

### Gas detection

Gas chromatography analyses of gas sampled from the headspace during the electrolysis were performed with an Agilent Technologies 7820A GC system equipped with a thermal conductivity detector. CO and H_2_ production was quantitatively detected using a CP-CarboPlot P7 capillary column (27.46 m in length and 25 μm internal diameter). Temperature was held at 150 °C for the detector and 34 °C for the oven. The carrier gas was argon flowing at 9.5 mL/min at constant pressure of 0.4 bars. Injection was performed via a 250-μL gas-tight (Hamilton) syringe previously degassed with CO_2_. Conditions allowed detection of both H_2_, O_2_, N_2_, CO, and CO_2_. Calibration curves for H_2_ and CO were determined separately by injecting known quantities of pure gas.

### Material characterizations

An X-Ray Photoelectron Spectrometer THERMO-VG ESCALAB 250 (RX source K AI (1486.6 eV)) was used. X-ray absorption spectra were collected at the LUCIA beamline of SOLEIL with a ring energy of 2.75 GeV and a current of 490 mA. The energy was monochromatized by means of a Si (111) double crystal monochromator. Data were collected in a primary vacuum chamber as fluorescence spectra with an outgoing angle of 5° using a Bruker silicon drift detector. The data were normalized to the intensity of the incoming incident energy and processed with the Athena software from the IFEFFIT package. For the EXAFS analysis, an *E*_0_ value of  7715.4 eV was used for the cobalt K-edge jump energy. SEM using a field emission gun was performed using a Zeiss Supra 40. Infrared spectra (IR) were recorded on a Bio-Rad FTS 175 C Fourier transform infrared spectrometer spectrophotometer. Ultraviolet (UV)–visible absorption spectra were obtained using a Shimadzu 2001 UV spectrophotometer. High resolution mass spectra were measured on an Agilent 6530 Accurate-Mass Q-TOF LC/MS spectrometer equipped with electrospray ionization source. NMR spectra were recorded in deuterated chloroform (CDCl_3_) and THF-d_8_ on a Varian 500 MHz spectrometer. Melting points were recorded on a Stuart SMP apparatus.

## Supplementary information

Supplementary Information

## Data Availability

Data supporting the findings of this study are available within the Article and its Supplementary Information, or from the corresponding authors upon reasonable request.
