# Supplementary material for: CO2 electrochemical catalytic reduction with a highly active cobalt phthalocyanine
Source: Nat Commun. 2019 Aug 9;10:3602. doi: 10.1038/s41467-019-11542-w (PMC6689005; doi:10.1038/s41467-019-11542-w)
Supplement: Supplementary file 1 — Supplementary Information [file 41467_2019_11542_MOESM1_ESM.pdf]

# $CO_2$ electrochemical catalytic reduction with a highly active cobalt phthalocyanine

Wang et al.

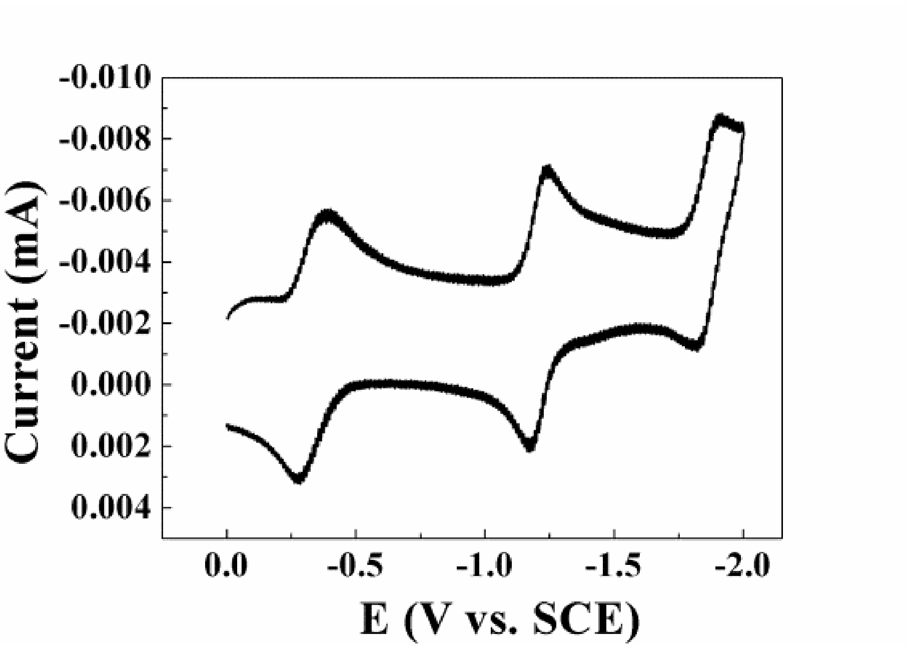


**Supplementary Figure 1 Cyclic voltammogram of CoPc2 in DMSO solution. The CV data were recorded at** *v* = 0.1 V $s^{-1}$of **CoPc2** (1 mM) at a glassy carbon electrode (*d* = 3 mm) in DMSO + 0.1 M $nBu_4NPF_6$ under Ar. Potentials are referred to the SCE reference electrode.

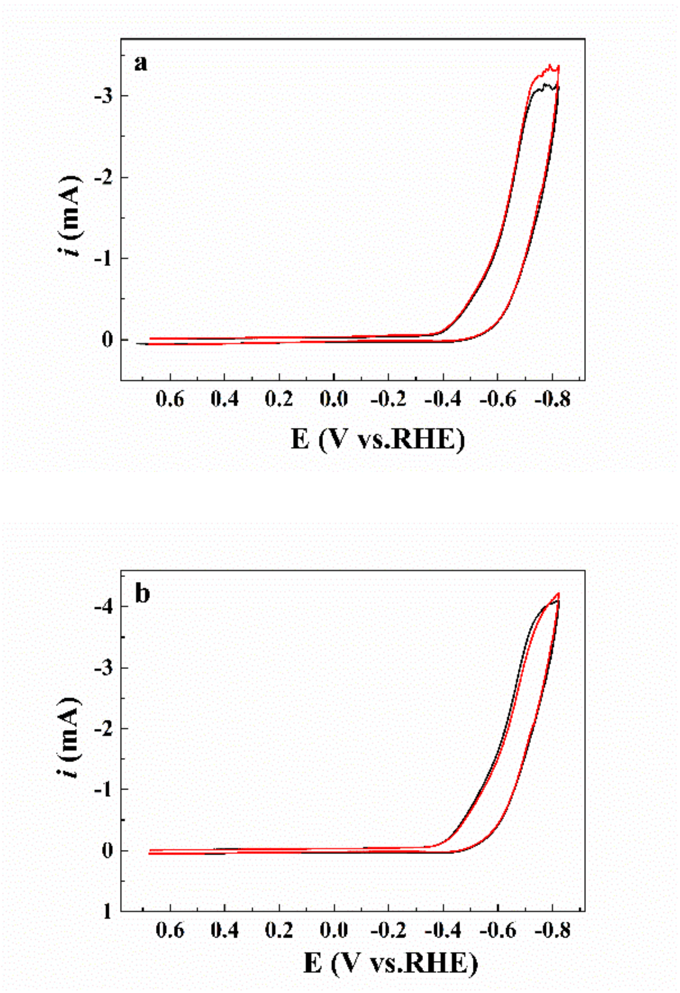


**Supplementary Figure 2 Repetitive cyclic voltammetries of the hybrid systems.** (a) **CoPc1**@MWCNTs and (b) **CoPc2**@MWCNTs films deposited onto a glassy carbon electrode (*d* = 3 mm) in 0.5 M $NaHCO_3$ solution saturated with $CO_2$ (pH 7.3; black, $1^{st}$ scan; red, $2^{nd}$ scan), at $v$ = 0.1 V $s^{-1}$.

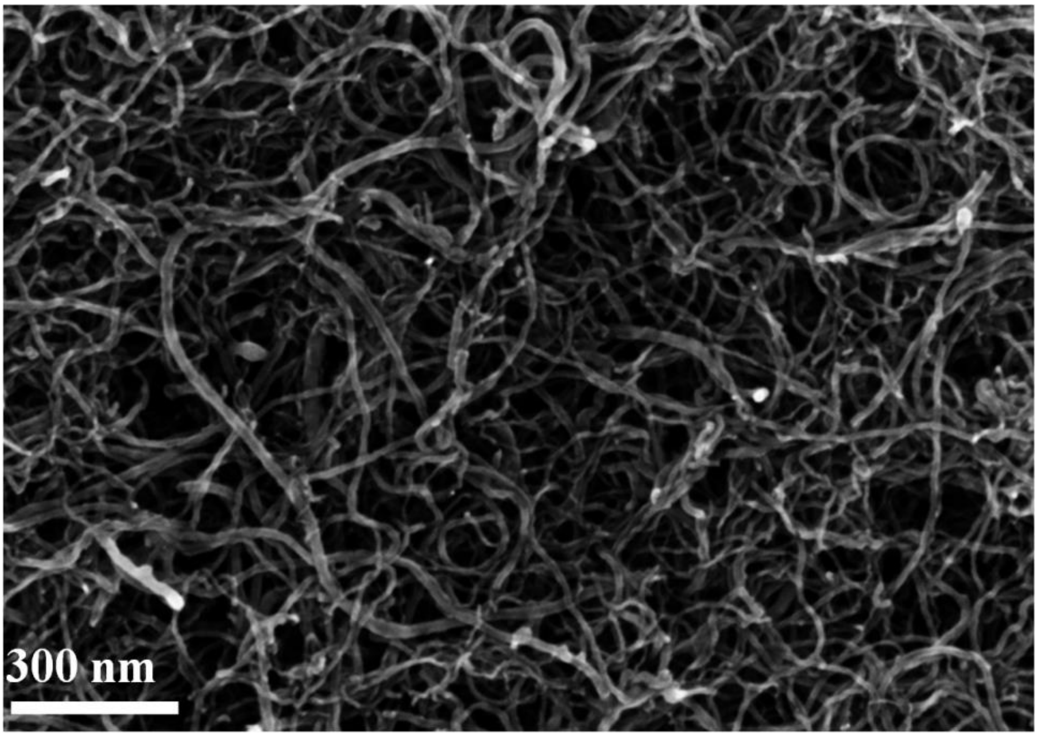


**Supplementary Figure 3 SEM image of a catalytic film deposited onto carbon paper.** The smooth sidewalls and the lower catalyst loading ($\Gamma_{\mathbf{(CoPc2)}}$ = 14.4 nmol cm$^{-2}$ (total amount of added catalysts)) as compared to manuscript reference 28 does suggest that agglomeration of the catalyst is unlikely.

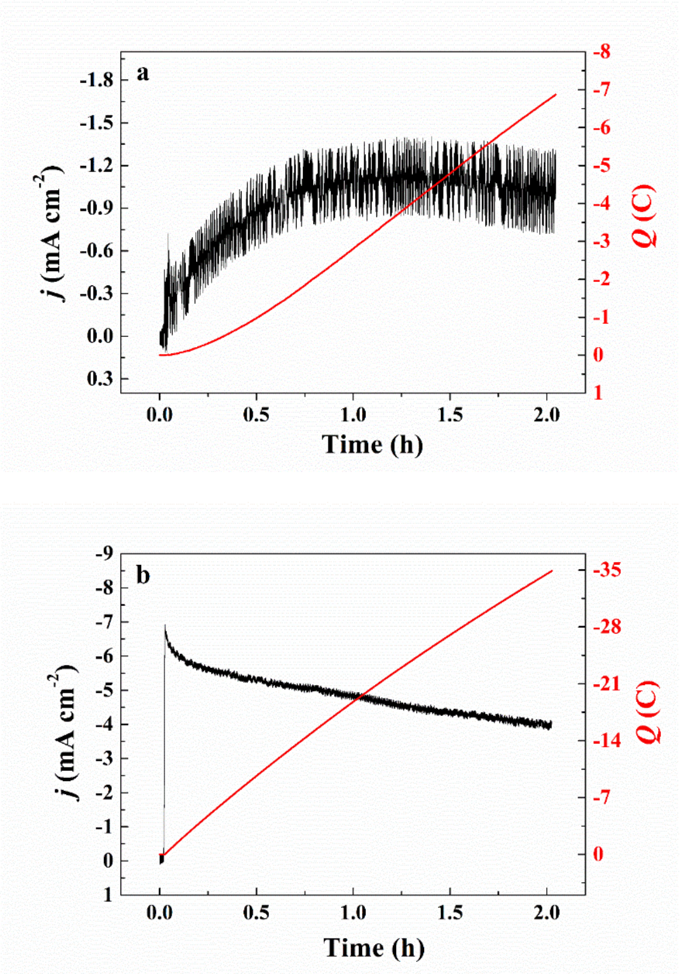


**Supplementary Figure 4 Blank electrolysis without the Co complex in the deposited film or under Ar atmosphere.** (a) At $E$ = -0.676 V vs. RHE with MWCNTs film in the absence of catalyst in $CO_2$ saturated 0.5 M $NaHCO_3$ solution (pH =7.3). (b) At $E$ = -0.605 V vs. RHE with **CoPc2**@MWCNTs (1:15) in Ar saturated 0.5 M $NaHCO_3$ solution (pH =8.5). In both cases, only $H_2$ was detected as reaction product.

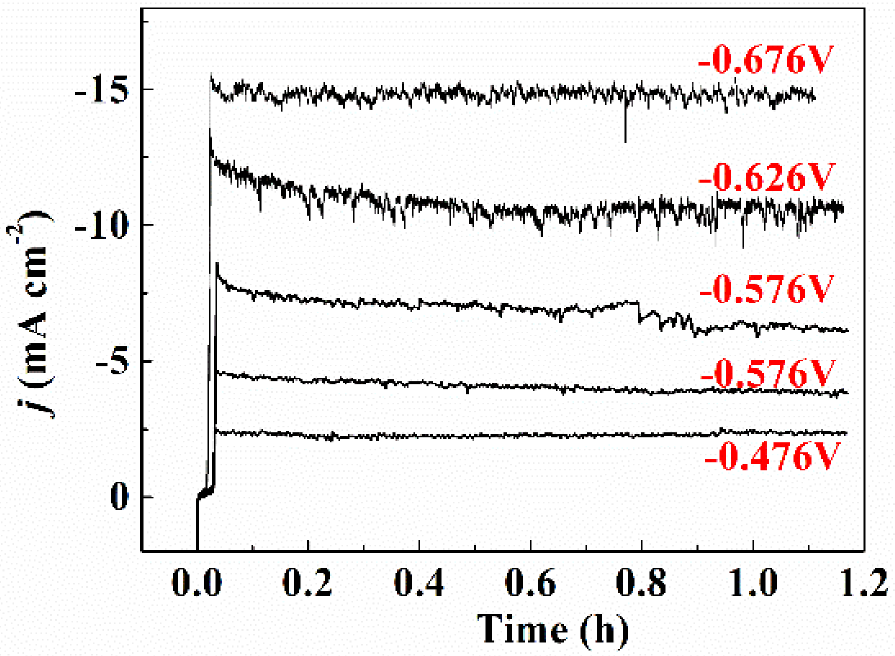


**Supplementary Figure 5 Controlled potential electrolysis of $CO_2$ reduction.** Variation of the total current density as a function of the electrolysis potential at optimized mass ratio for **CoPc1**@MWCNTs in a $CO_2$ saturated solution containing 0.5 M $NaHCO_3$ (pH 7.3).

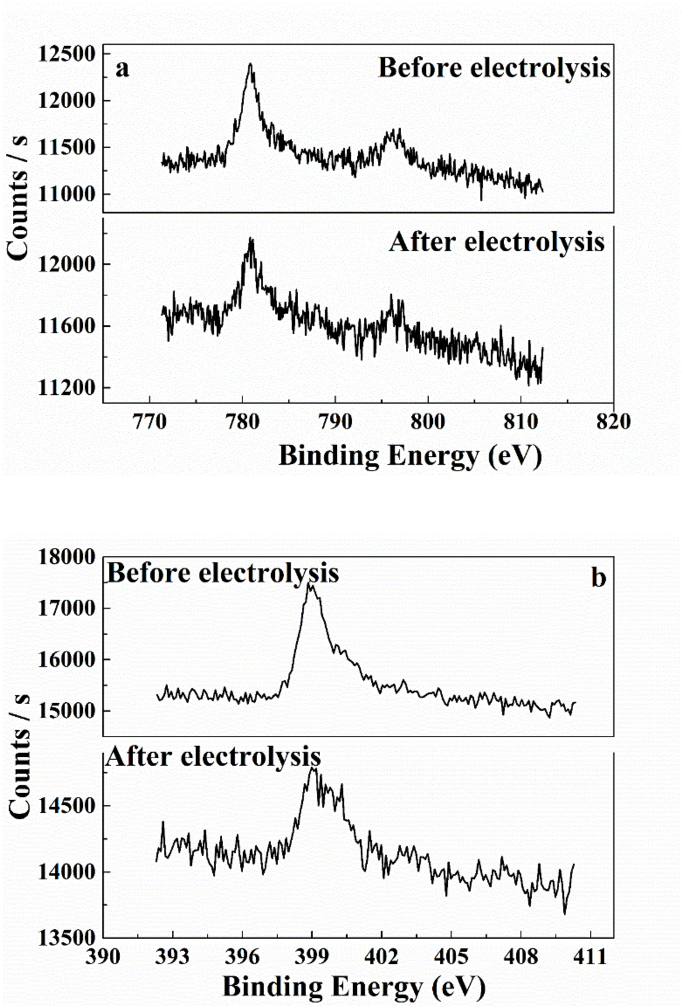


**Supplementary Figure 6 X-ray photoelectron spectroscopy characterization.** (a) Co 2p and (b) N 1s XPS spectra before and after a 7h electrolysis ($E$ = -0.676 V vs. RHE) with **CoPc2**@MWCNTs with a 1:15 ratio in a $CO_2$ saturated solution with 0.5 M $NaHCO_3$ (pH 7.3).

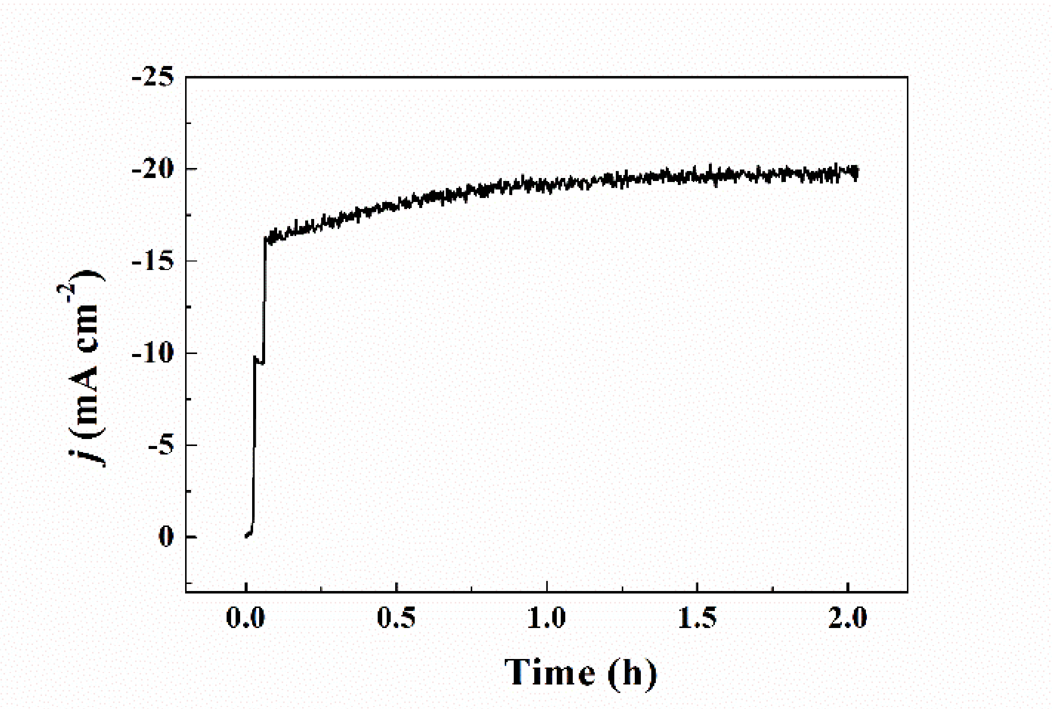


**Supplementary Figure 7 Controlled potential electrolysis of CoPc2 catalyst in acidic solution.** Bulk electrolysis at $E$ = - 0.971 V vs. RHE of a $CO_2$ saturated solution containing 0.5 M KCl (pH 4) using **CoPc2**@MWCNTs as catalyst.

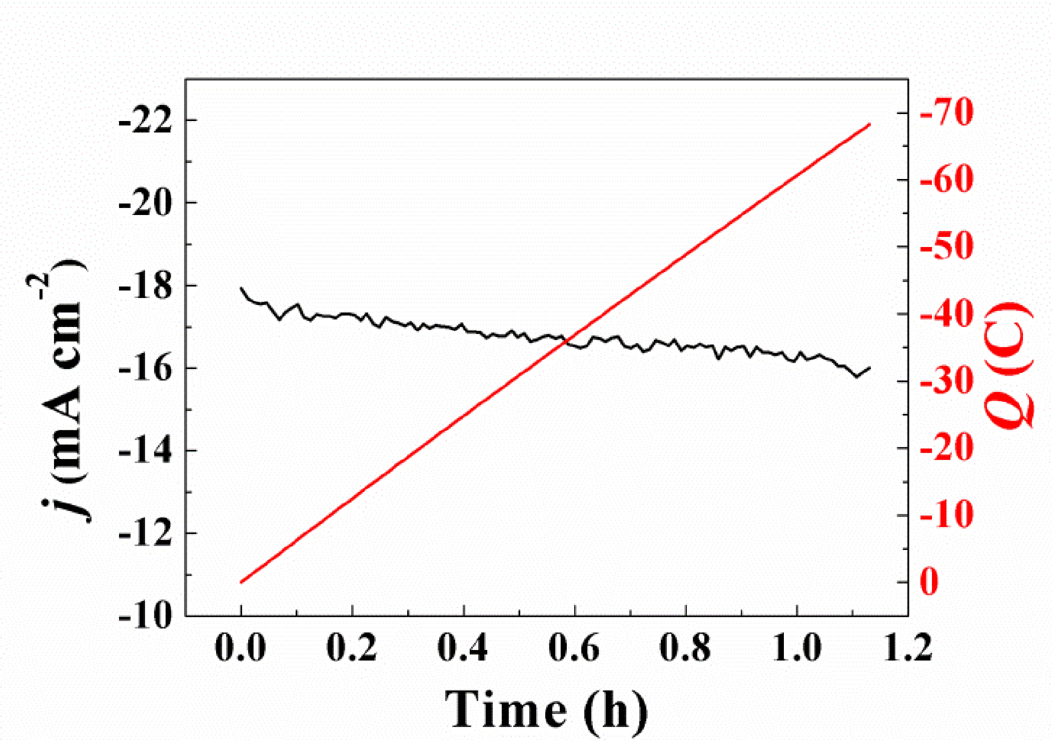


**Supplementary Figure 8 Electrolysis performance of 4-Co catalyst.** Bulk electrolysis at *E* = - 0.676 V vs. RHE of a $CO_2$ saturated solution containing 0.5 M $NaHCO_3$ (pH 7.3) using **4-Co**@MWCNTs as catalyst.

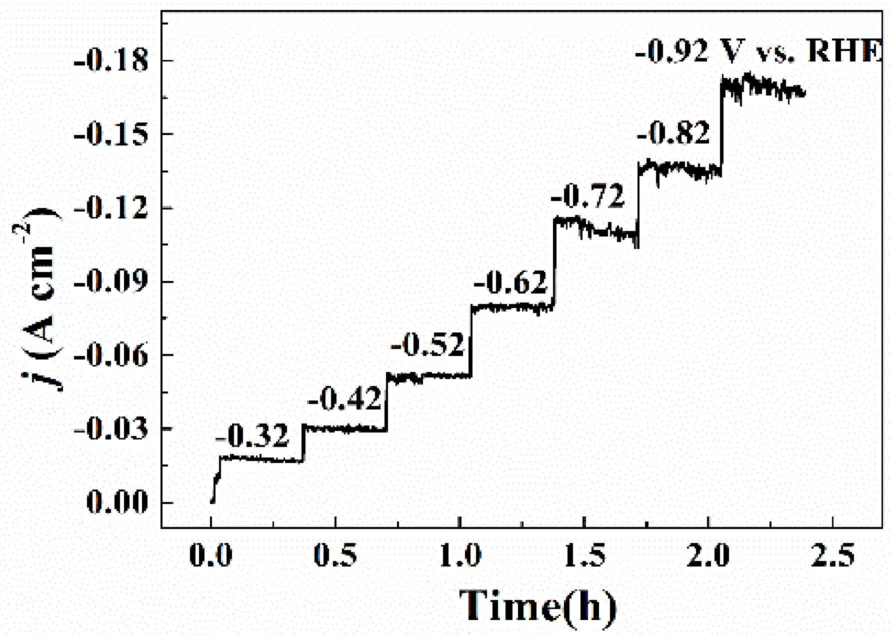


**Supplementary Figure 9 Controlled potential electrolysis of CoPc2 catalyst in basic solution.** Total current density as a function of the applied potential for **CoPc2**@carbon black deposited onto a carbon paper as cathodic catalytic material, in 1 M KOH solution (flow cell device, see main text). Each potential step was held for a duration of 20 min.

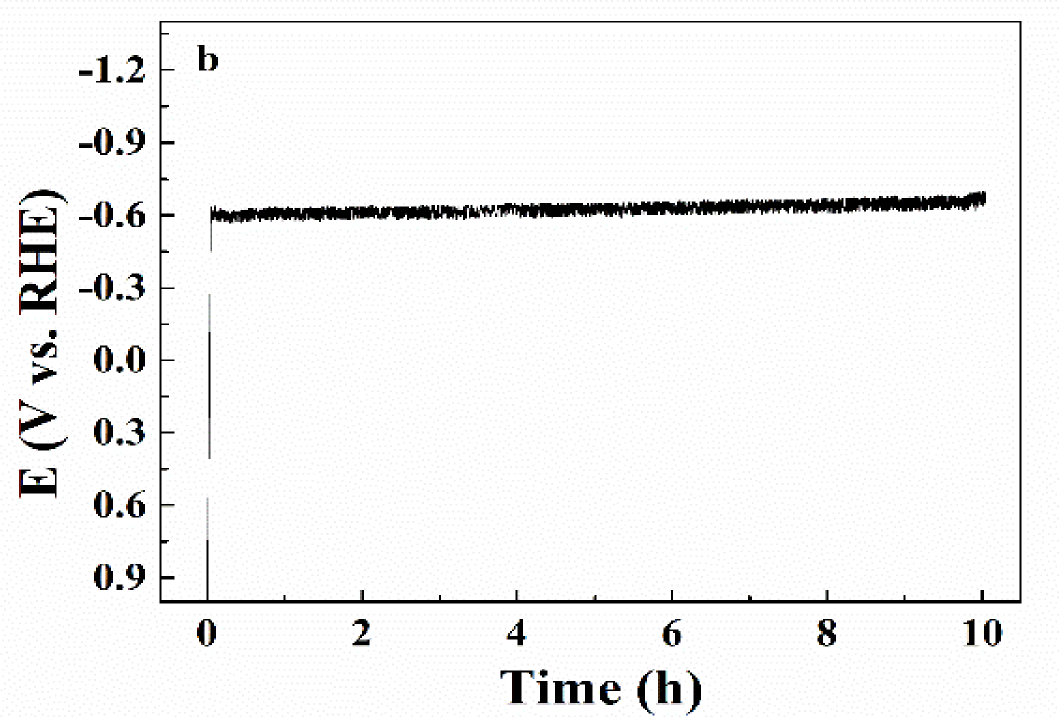


**Supplementary Figure 10 Reduction potential as a function of time for $CO_2$RR electrolysis.** Bulk electrolysis at fixed current density (75 mA $cm^{-2}$) with **CoPc2**@carbon black deposited onto a carbon paper as cathodic catalytic material, in 1 M KOH solution (flow cell device, see main text). During the electrolysis, 2.5 g KOH was added every half hour into the cathode solution to maintain the pH balance.

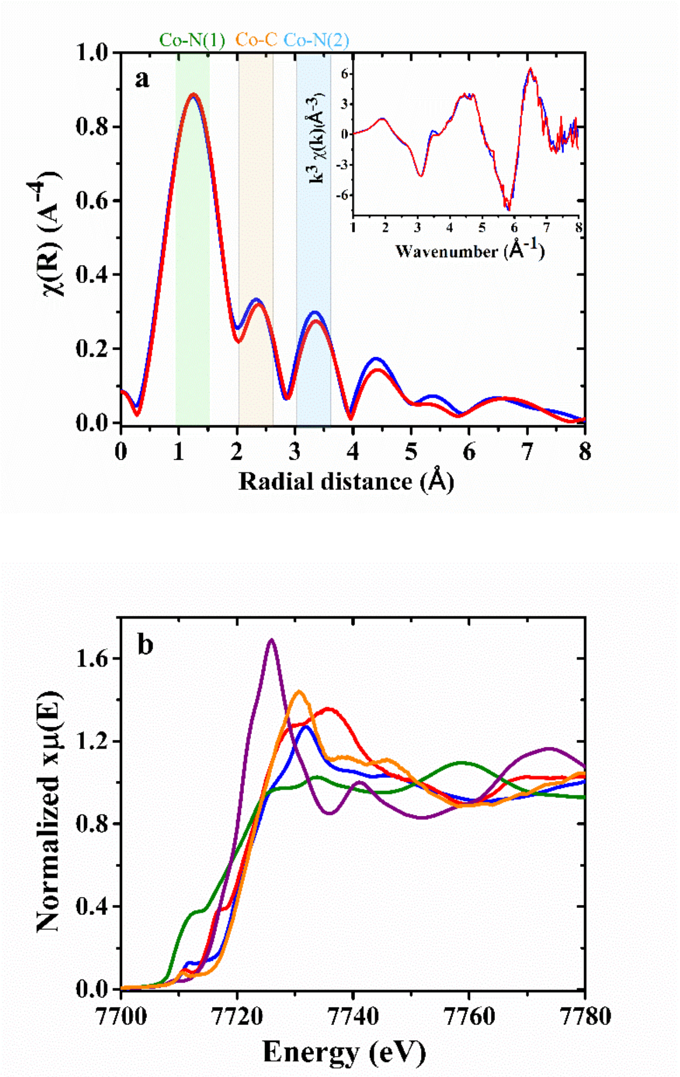


**Supplementary Figure 11 X-ray absorption spectroscopy characterizations of catalyst and reference chemicals.** (a) K-space (insert) and Fourier transform EXAFS spectra of **CoPc2**@carbon black before (blue) and after electrolysis (*E* = -0.72 V vs RHE) (red) in 1 M KOH solution. (b) Co K-edge XANES spectra of the **CoPc2**@carbon black electrode after electrolysis (*E* = -0.72 V vs RHE) (red) together with those of reference compounds CoO (purple), CoOOH (orange), $Co_3O_4$ (blue) and metallic Co (green).

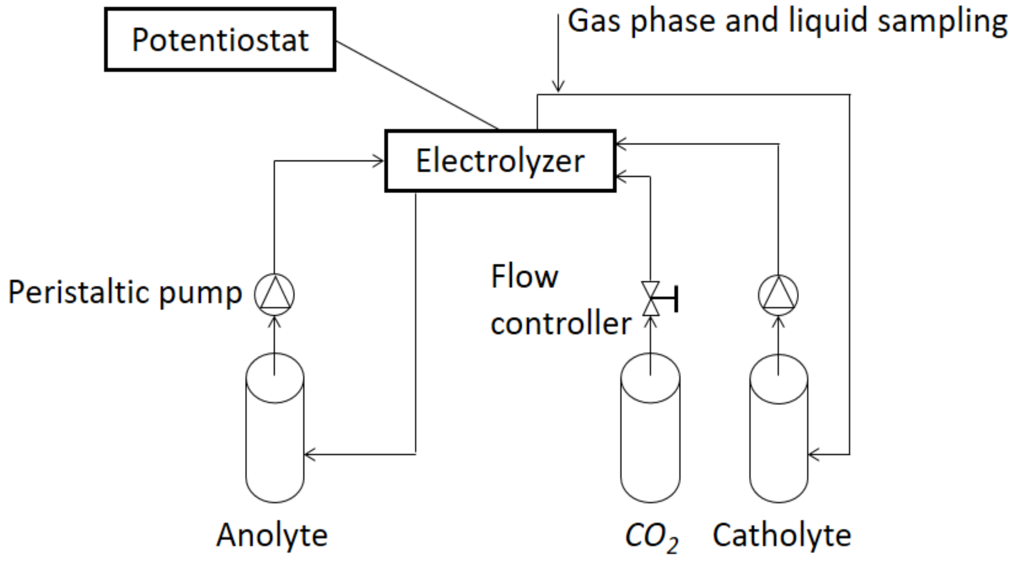


**Supplementary Figure 12 Set-up with flow cell electrolyzer.**

## The pathway to synthesize phthalonitrile and phthalocyanine catalysts.

Cobalt catalyst was prepared as illustrated on Supplementary Figure 13. Characterizations of Phthalonitrile **1**, Phthalocyanines **3**, **5** and **CoPc2** are provided in Supplementary Figures 14-27.

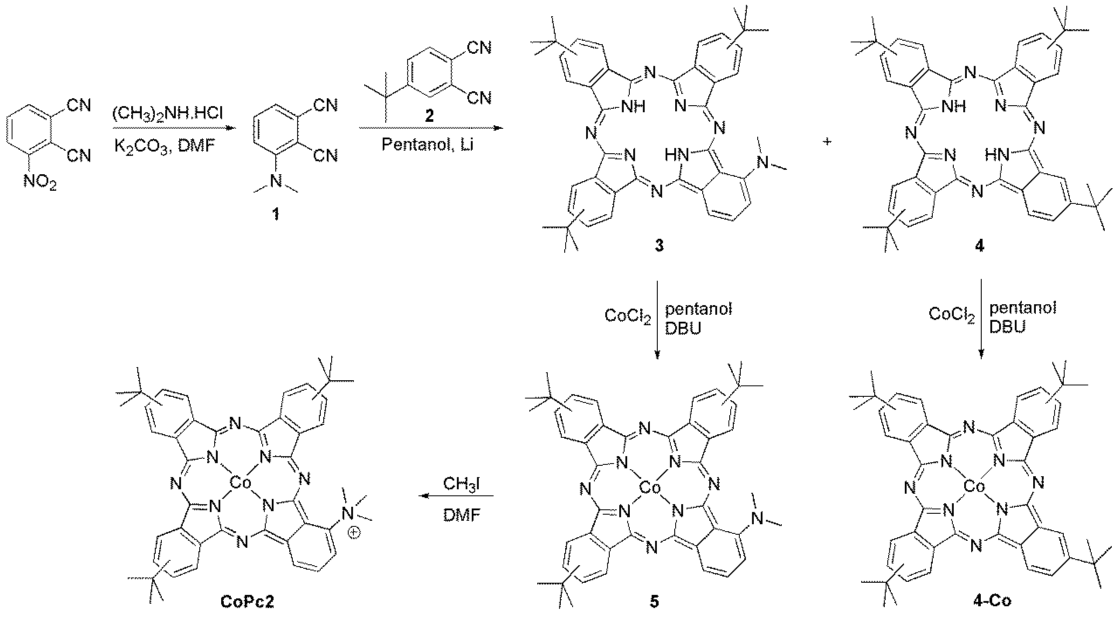


**Supplementary Figure 13 Synthesis of phthalonitrile and phthalocyanines.**

Synthesis of 3-(dimethylamino)phthalonitrile **1**

3-Nitrophthalonitrile (2 g, 11.5 mmol) and dimethylamine hydrochloride (2.8 g, 34.6 mmol) were dissolved in anhydrous DMF (30 mL) under argon atmosphere then finely powdered dry potassium carbonate (30 g, 217.5 mmol) was added portion-wise over 15 min. The reaction mixture was stirred under argon at 65 °C for 24 h then poured into water (250 mL). The resulting solid was collected by filtration and washed with water. After drying in vacuum, the crude product was recrystallized from ethanol. Yield: 80 % (1.57 g). m.p. 105 °C. $^1$H NMR (500 MHz, $CDCl_3$): δ, ppm 3.18 (s, 6H), 7.12 (d, 1H), 7.15 (d, 1H), 7.46 (t, 1H). $^{13}$C NMR (125 MHz, $CDCl_3$): δ, ppm 172.4, 155.4, 133.1, 123.0, 120.5, 118.0, 116.6, 116.3, 110.0, 100.6, 42.7. FT-IR (ν, $cm^{-1}$): 2992, 2937, 2874, 2203, 1584, 1486, 1425, 1357, 1244, 1192, 1122, 1008, 792, 722.

Synthesis of phthalocyanine **3**

Granules of lithium were added to anhydrous *n*-pentanol (10 mL). This mixture was heated to 60 °C under argon flux until total consumption of the granules. 3-(dimethylamino)phthalonitrile **1** (0.25 g, 1.46 mmol) and 4-*tert*-butylphthalonitrile **2** (3.2 g,

17.52 mmol) were then added and this reaction mixture was refluxed for 18 h, then cooled to room temperature and poured to an ethanol/water mixture. The resulting dark blue-green precipitate was filtered off, washed several times with water and dried. Phthalocyanine **3** was isolated from this crude mixture of phthalocyanines by chromatography on silica gel using a mixture of $CH_2Cl_2$/EtOH (100:1) as the eluent. The tetra-*tert*-butylphthalocyanine **4** was first eluted and the phthalocyanine **3** was the second eluted compound. Yield: 15 % (160 mg). $^1$H NMR (500 MHz, THF-$d_8$): δ, ppm -2.04 (s, 1H), -1.95 (s, 1H), 1.84 (m, 10H), 1.88-1.94 (m, 17H), 3.70 (s, 3H), 7.45 (d, 1H), 7.79 (m, 1H), 8.10-8.40 (m, 4H), 8.61 (d, 1H), 8.85-8.91 (m, 1H), 8.98- 9.24 (m, 4H), $^{13}$C NMR (125 MHz, THF-$d_8$): δ, ppm 152.97, 152.94, 152.88, 152.84, 152.61, 152.58, 150.16, 150.12, 133.67, 129.92, 127.43, 127.30, 127.22, 126.75, 125.50, 122.12, 122.07, 121.93, 121.90, 121.77, 118.69, 118.65, 118.51, 118.48, 118.41, 117.87, 117.83, 114.74, 78.54, 78.28, 78.02, 44.36, 39.98, 37.15, 37.06, 35.67, 35.60, 35.50, 31.89, 31.43, 31.32, 29.65, 29.32, 22.58, 13.43. ESI-HRMS: m/z 726.4030 $[M]^+$ calculated for $C_{46}H_{47}N_9$: 725.95. UV-vis (DMF): $\lambda_{max}$ nm (log ε) 346 (3.90), 689 (4.87), 718 (5.38). FT-IR (ν, cm$^{-1}$): 3288, 2955, 2863, 2776, 1618, 1501, 1316, 1186, 1014, 828, 742.

Synthesis of phthalocyanine **5**

A mixture of phthalocyanine **3** (50 mg, 0.06 mmol), $CoCl_2$ (18 mg, 0.12 mmol), and DBU (1 mL) in dried *n*-pentanol (5 mL) was heated to reflux for 18 h under argon. After cooling to room temperature, the reaction mixture was poured into an ethanol/water mixture. The resulting precipitate was filtered off and washed several times with water. Phthalocyanine **5** was purified by chromatography on silica gel using a mixture of $CH_2Cl_2$/EtOH (50:1) as the eluent. Yield: 75 % (35 mg). ESI-HRMS: m/z 782.3234 $[M]^+$ calculated for $C_{46}H_{45}CoN_9$: 782.86. UV-vis (DMF): $\lambda_{max}$ nm (log ε) 332 (3.70), 693 (4.74); FT-IR (ν, cm$^{-1}$): 2955, 2856, 1606, 1457, 1316, 1254, 1094, 804, 737.

Synthesis of phthalocyanine **CoPc2**

Phthalocyanine **5** (35 mg, 0.044 mmol) was dissolved in DMF (5 mL), and methyl iodide (0.2 g, 1.5 mmol) was added. The mixture was stirred at room temperature for 16 h then poured into diethyl ether (25 mL). The resulting blue precipitate was filtered off, washed with ether and dried. Yield: 32 mg (88%). ESI-HRMS: m/z 797.3668 $[M]^+$ calculated for $C_{47}H_{48}CoN_9$: 797.90. Anal. calcd for $C_{47}H_{58}CoIN_9O_5$ (CoPc2.5$H_2O$): C, 55.28; H, 5.98; N, 11.88. Found: C, 55.62; H, 5.76; N, 12.42. UV-vis (DMF): $\lambda_{max}$ nm (log ε) 326 (4.41), 664 (4.65). FT-IR (ν, cm$^{-1}$) 3047, 2949, 2851, 1655, 1606, 1476, 1328, 1254, 1088, 933, 829, 749.

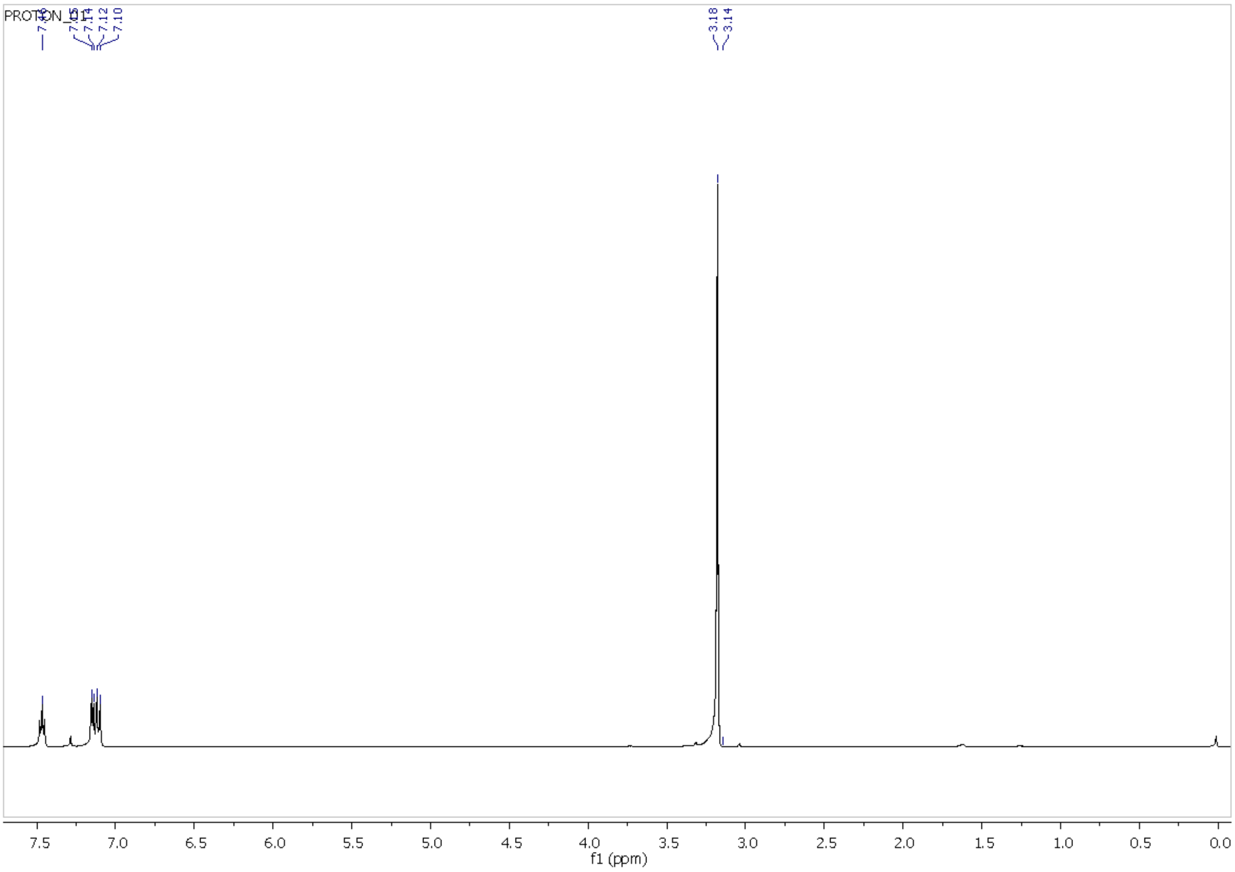


**Supplementary Figure 14 $^{1}H$ NMR spectrum of phthalonitrile 1 ($CDCl_3$).**

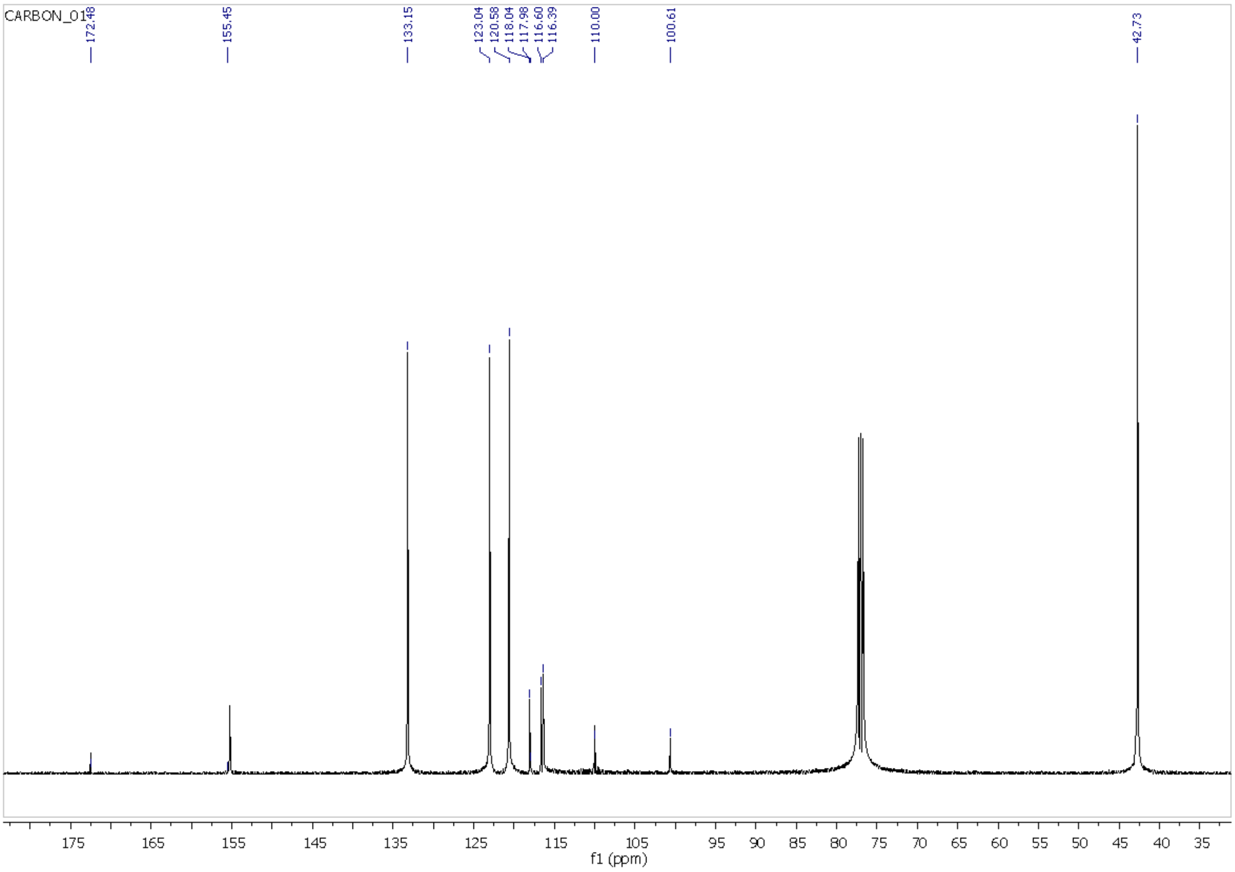


**Supplementary Figure 15 $^{13}$C NMR spectrum of phthalonitrile 1 ($CDCl_3$).**

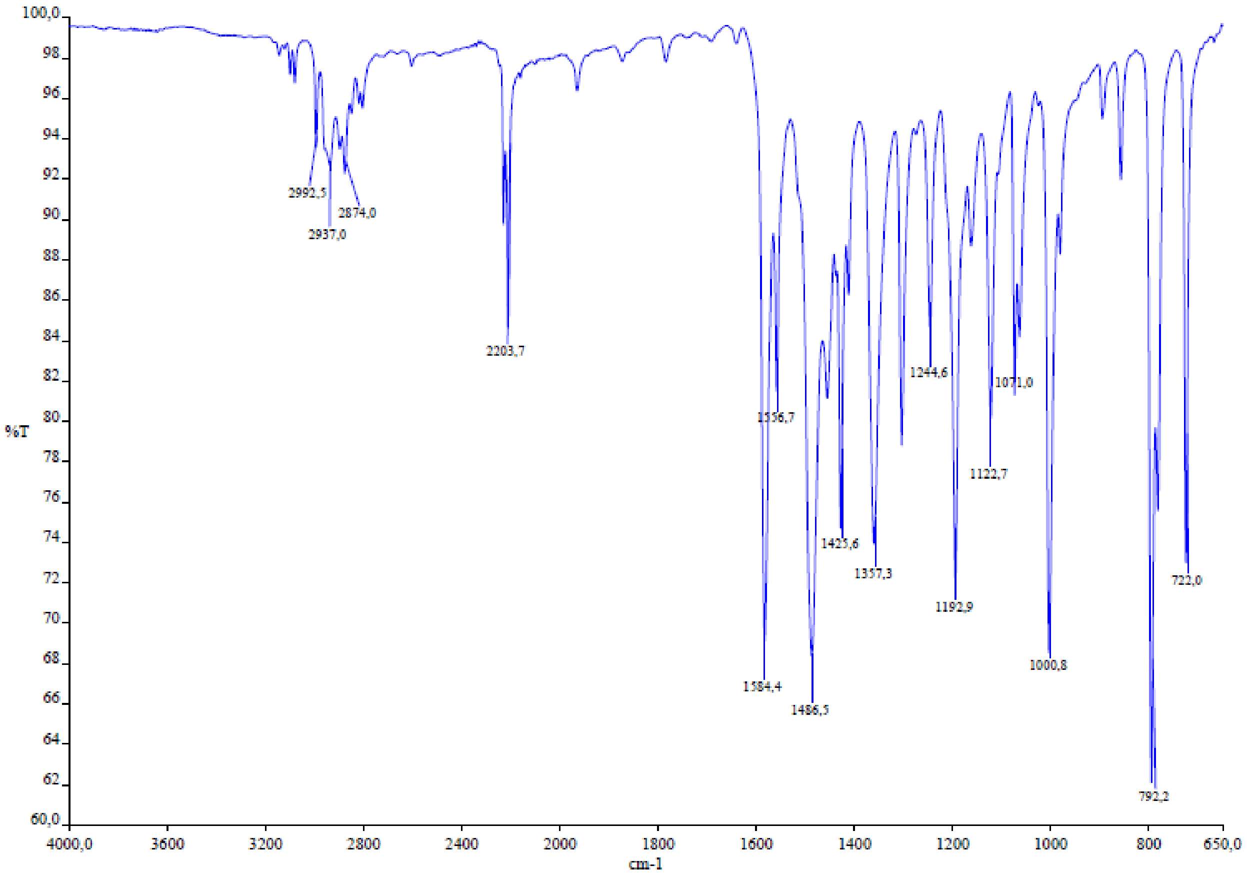


**Supplementary Figure 16 FT-IR spectrum of phthalonitrile 1.**

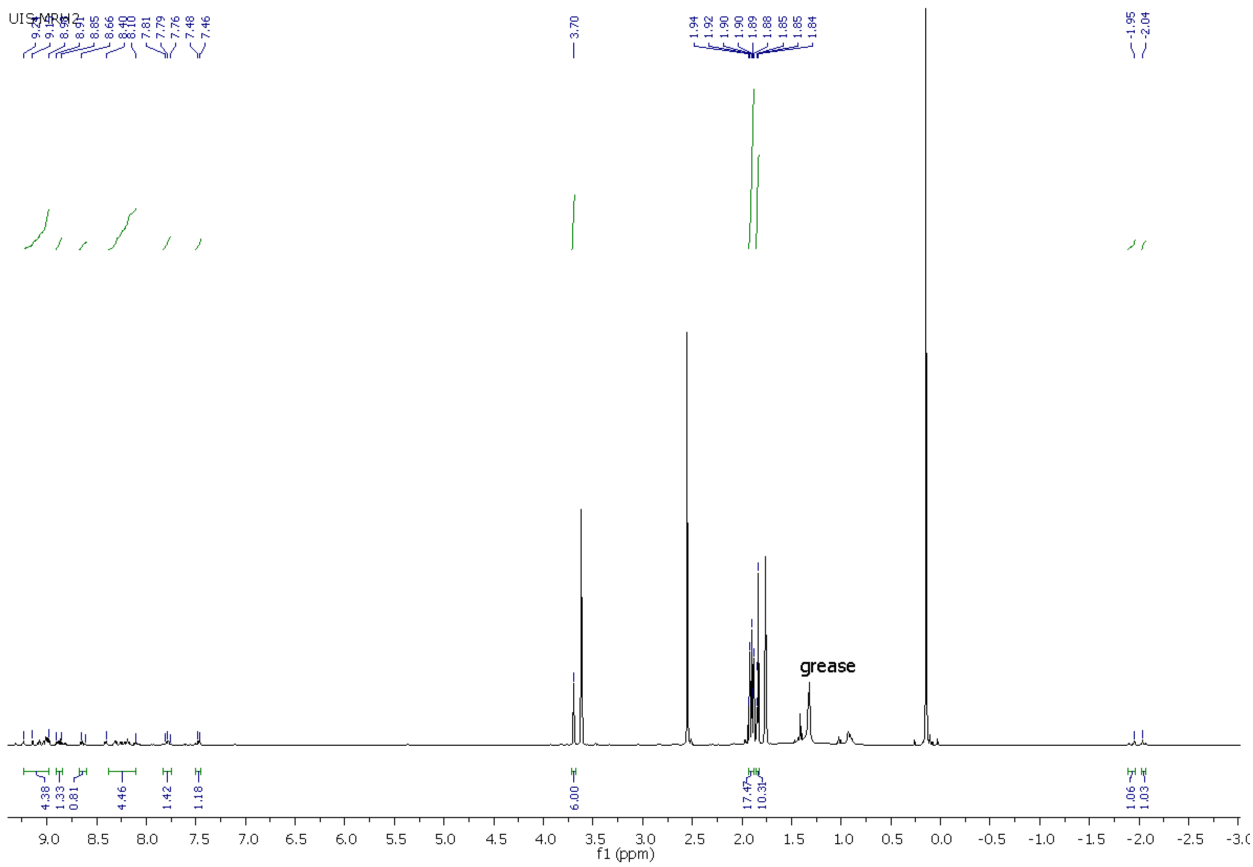


**Supplementary Figure 17 $^{1}$H NMR spectrum of phthalocyanine 3 (THF-$d_8$).**

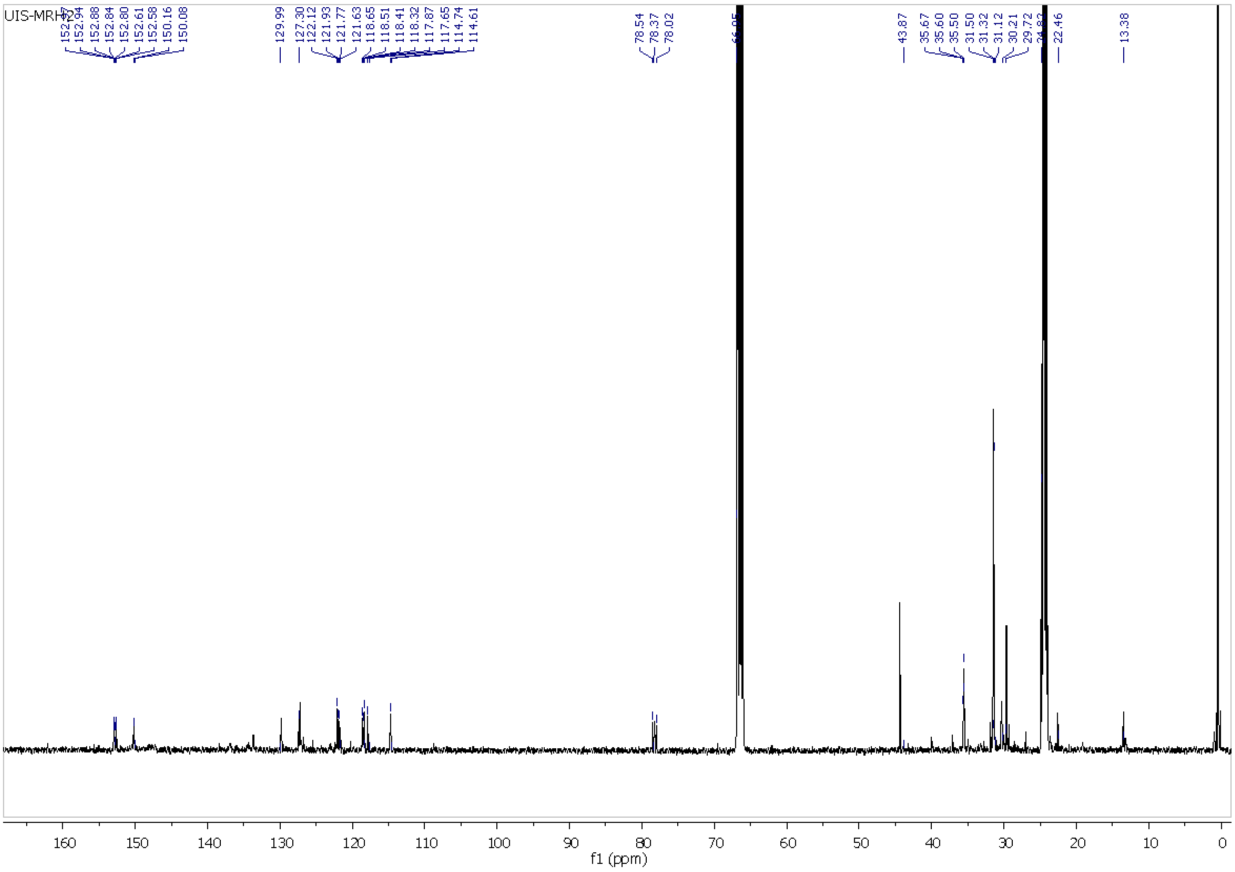

**Supplementary Figure 18 $^{13}C$ NMR spectrum of phthalocyanine 3 (THF-$d_8$).**

## User Spectra

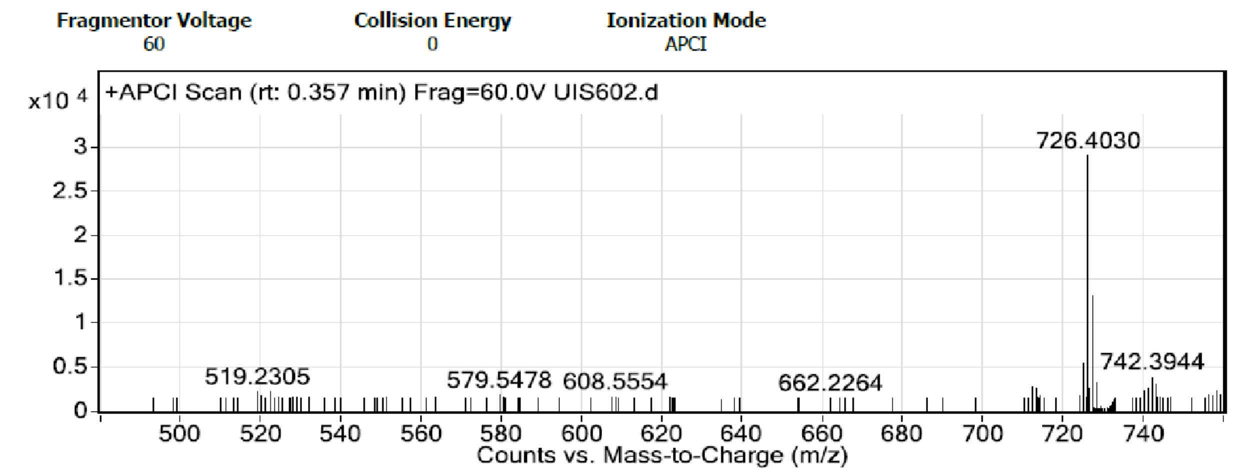


**Supplementary Figure 19 HR-MS spectrum of phthalocyanine 3.**

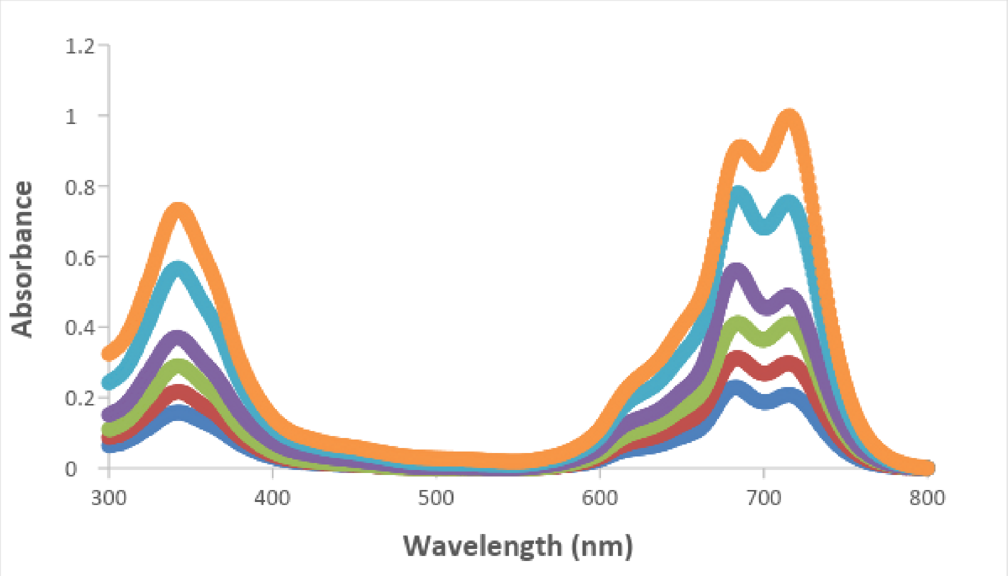


**Supplementary Figure 20 UV-vis spectrum of phthalocyanine 3 in DMF at 2, 4, 6, 8, 10 and 12 × $10^{-6}$ M.**

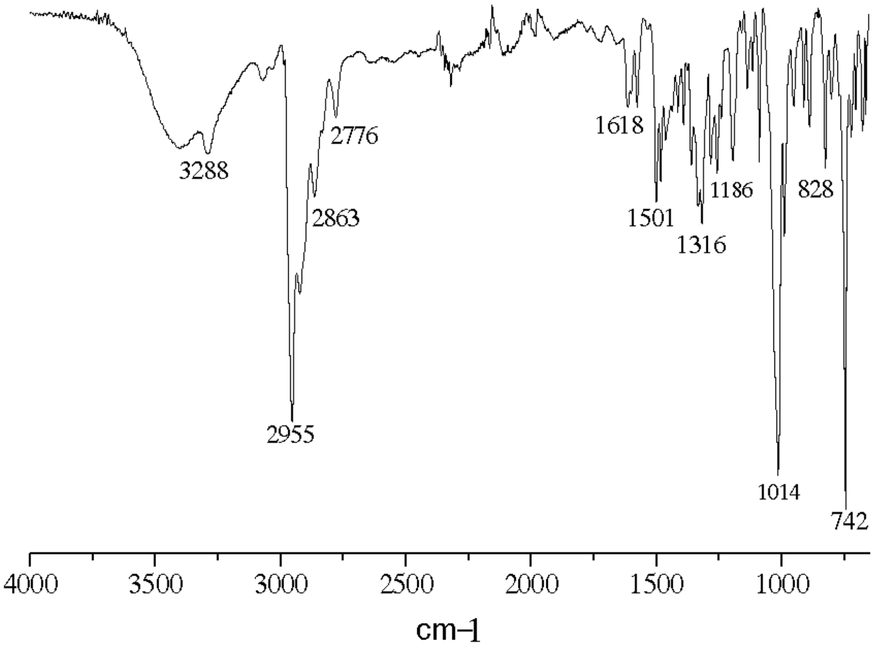


**Supplementary Figure 21 FT-IR spectrum of phthalocyanine 3.**

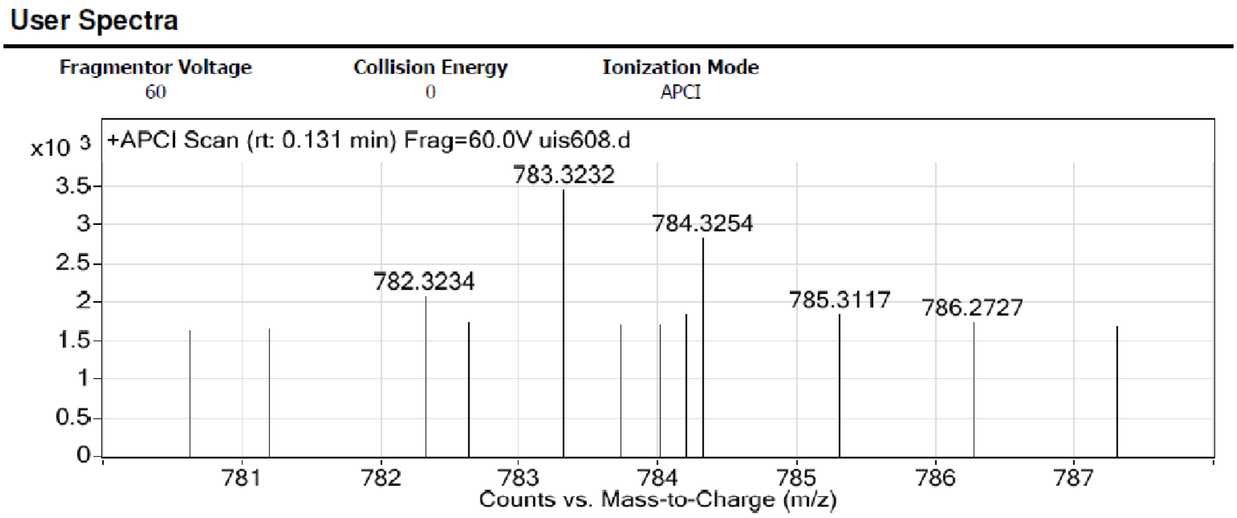


**Supplementary Figure 22 HRMS spectrum of phthalocyanine 5.**

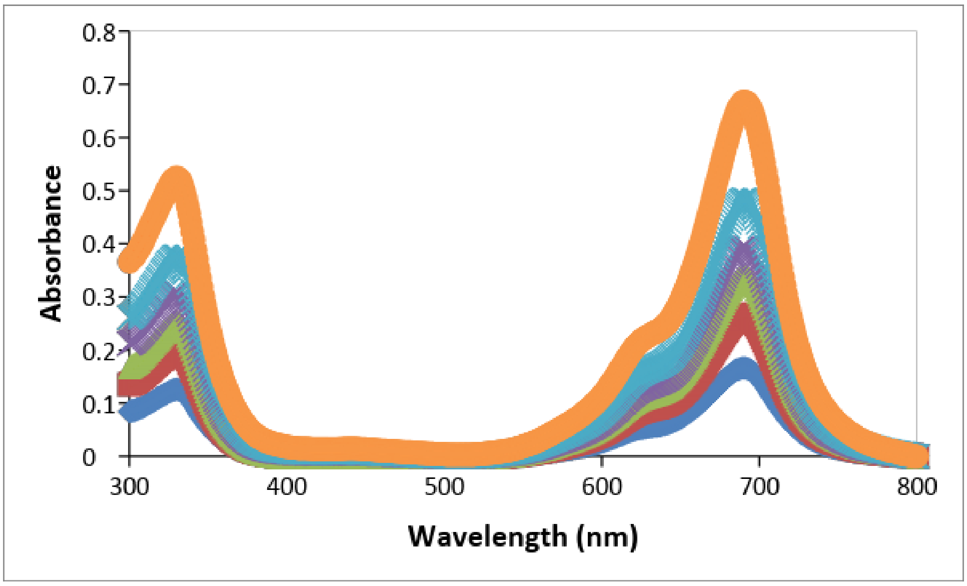


**Supplementary Figure 23 UV-vis spectrum of phthalocyanine 5 in DMF at 2, 4, 6, 8, 10 and 12 × $10^{-6}$ M.**

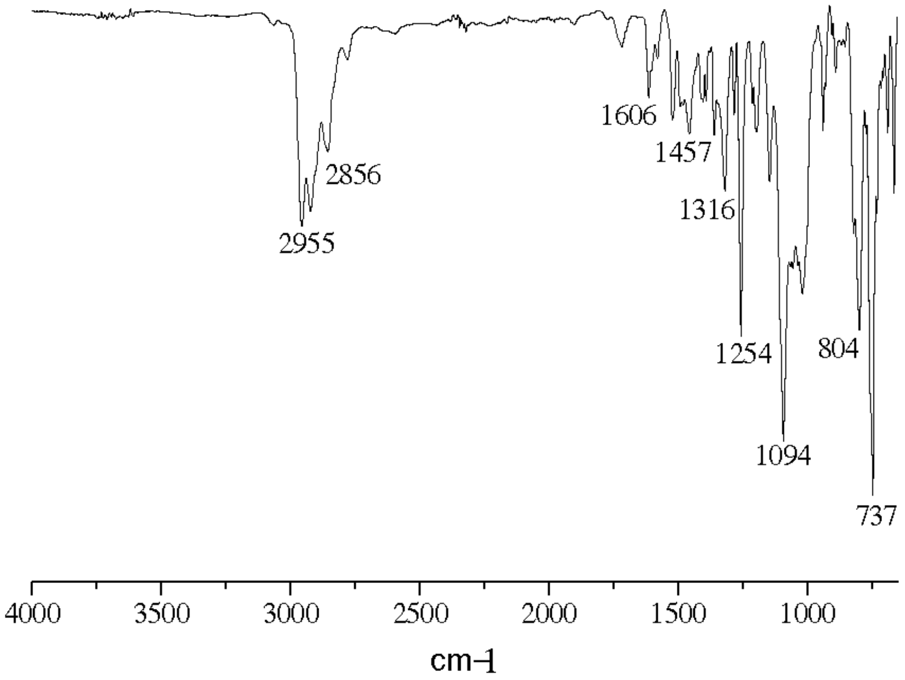


**Supplementary Figure 24 FT-IR spectrum of phthalocyanine 5.**

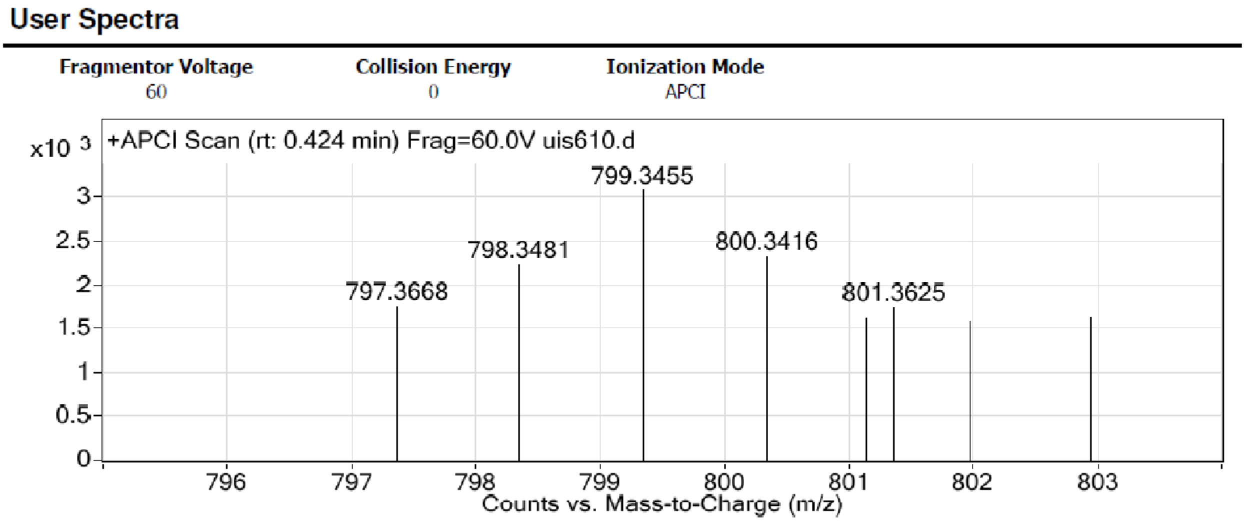


**Supplementary Figure 25 HR-MS spectrum of phthalocyanine CoPc2.**

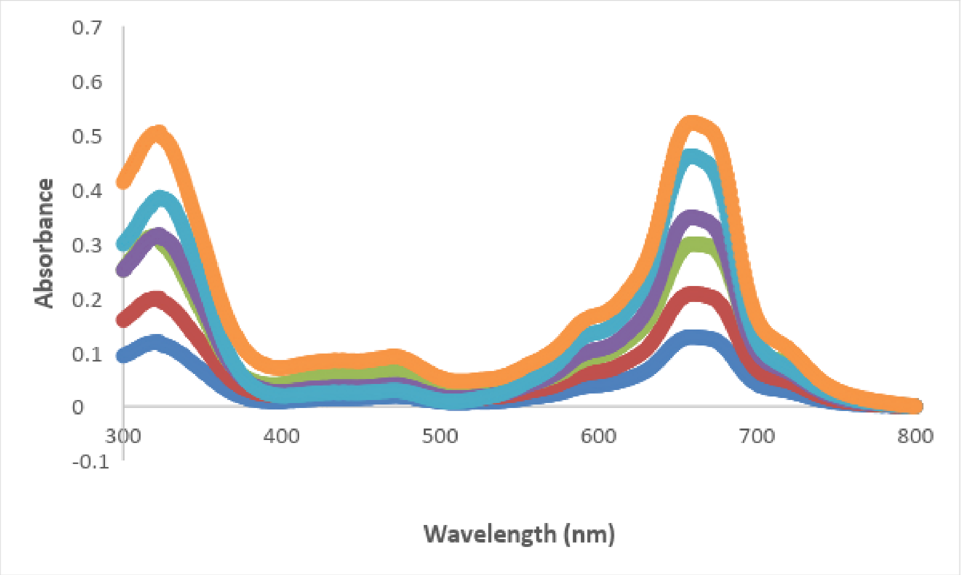


**Supplementary Figure 26 UV-vis spectrum of phthalocyanine CoPc2 in DMF at 2, 4, 6, 8, 10 and 12 × $10^{-6}$ M.**

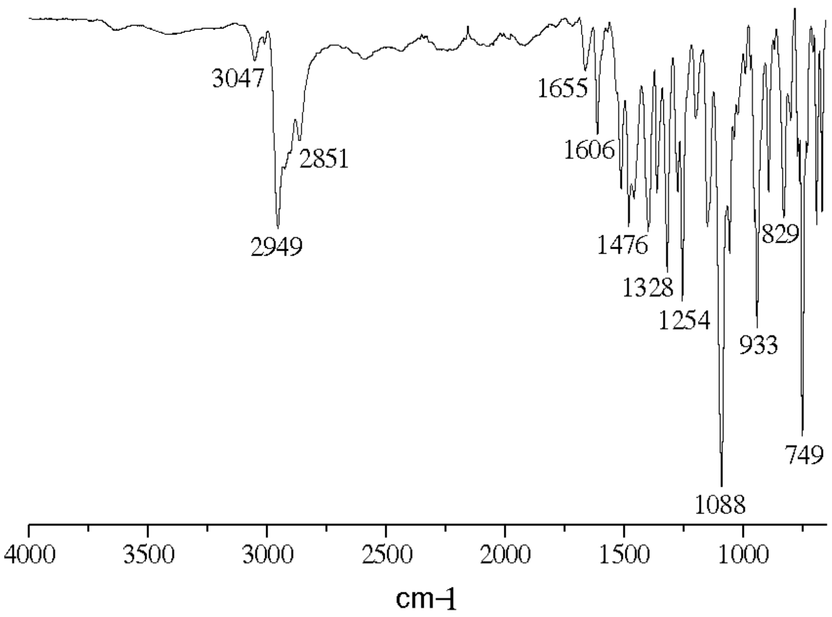


**Supplementary Figure 27 FT-IR spectrum of phthalocyanine CoPc2.**
